# Identification and characterization of *Brevibacillus halotolerans* B-4359: a potential antagonistic bacterium against red pepper anthracnose in Korea

**DOI:** 10.3389/fmicb.2023.1200023

**Published:** 2023-06-19

**Authors:** Heejin Kim, Younmi Lee, Ye-Ji Hwang, Mi-Hwa Lee, Kotnala Balaraju, Yongho Jeon

**Affiliations:** ^1^Department of Plant Medicals, Andong National University, Andong, Republic of Korea; ^2^Agricultural Science and Technology Research Institute, Andong National University, Andong, Republic of Korea; ^3^Microbiology Research Department, Nakdonggang National Institute of Biological Resources, Sangju, Republic of Korea

**Keywords:** phytopathogen, *Brevibacillus halotolerans* B-4359, *Colletotrichum acutatum*, red pepper anthracnose, sustainable agriculture, whole-genome sequencing, biocontrol agent

## Abstract

Our study aimed to identify potential biocontrol agents (BCAs) against major phytopathogens under *in vitro* conditions by screening the Freshwater Bioresources Culture Collection (FBCC), Korea. Of the identified 856 strains, only 65 exhibited antagonistic activity, among which only one representative isolation, *Brevibacillus halotolerans* B-4359 was selected based on its *in vitro* antagonistic activity and enzyme production. Cell-free culture filtrate (CF) and volatile organic compounds (VOCs) of B-4359 were shown to be effective against the mycelial growth of *Colletotrichum acutatum*. Notably, B-4359 was found to promote spore germination in *C. acutatum* instead of exhibiting a suppressive effect when the bacterial suspension was mixed with the spore suspension of *C. acutatum*. However, B-4359 showed an excellent biological control effect on the anthracnose of red pepper fruits. Compared to other treatments and untreated control, B-4359 played a more effective role in controlling anthracnose disease under field conditions. The strain was identified as *B. halotolerans* using BIOLOG and 16S rDNA sequencing analyses. The genetic mechanism underlying the biocontrol traits of B-4359 was characterized using the whole-genome sequence of B-4359, which was closely compared with related strains. The whole-genome sequence of B-4359 consisted of 5,761,776 bp with a GC content of 41.0%, including 5,118 coding sequences, 117 tRNA, and 36 rRNA genes. The genomic analysis identified 23 putative secondary metabolite biosynthetic gene clusters. Our results provide a deep understanding of B-4359 as an effective biocontrol agent against red pepper anthracnose for sustainable agriculture.

## Introduction

Plant pathogenic bacteria and fungi are responsible for major crop losses in terms of both the quality and quantity of agro-produce (Chakraborty and Newton, 2011). Approximately 10–15% of crop yield losses are caused by pathogenic infections globally (Peng et al., 2021). Phytopathogens produce toxins and enzymes that are harmful to host plants; even at low concentrations, these toxins are sufficient to destroy normal physiological functions in host plants (Thynne et al., 2017). Among bacterial phytopathogens, gram-negative bacteria in the genera, *Xanthomonas*, *Pectobacterium*, and *Erwinia* cause the most widespread and destructive losses in agricultural production (Mansfield et al., 2012).

Over the last two decades, the use of beneficial bacteria for the biological control of plant pathogens has become a major tool for protecting several crops (Samaras et al., 2021). Among several beneficial bacteria, species that can protect crop plants in several ways and promote plant growth either by direct or indirect mechanisms are commonly referred to as plant growth-promoting rhizobacteria (PGPR) (Gowtham et al., 2018). Plant diseases caused by fungi, bacteria, and viruses result in an estimated yield loss of 14% in diverse crops of agricultural importance, leading to an economic loss of USD 220 billion annually (Peng et al., 2021). Among these, plant pathogenic fungi are responsible for the majority of the devastating diseases, leading to famine and affecting food security worldwide (Fisher et al., 2012). Phytopathogenic fungi produce toxins that play a key role in the development of plant diseases, thereby adversely affecting host plants (Soyer et al., 2015). Many species of the genus *Colletotrichum* are associated with plant diseases commonly referred to as anthracnoses. *Colletotrichum* spp. can affect a wide range of hosts globally and cause crop loss in many staple foods (Prusky et al., 2000). Among the species of *Colletotrichum*, members of the *Colletotrichum acutatum* species complex have been well-documented causing diseases in a wide range of hosts (Talhinhas et al., 2005), including anthracnose in red pepper (Than et al., 2008).

Anthracnose in red peppers is associated with several *Colletotrichum* species, including *C. acutatum* (Harp et al., 2014), and may also infect other fruit and vegetable crops (Han et al., 2015). Red pepper anthracnose is typically characterized by dark brown to black, circular water-soaked spots with concentric rings of black acervuli developing beneath the skin of the fruit (Parisi et al., 2020); which often coalesce and cause softening and fruit rot (Montri et al., 2009). Anthracnose is typically controlled by the application of chemical fungicides under field conditions. The continuous use of chemical pesticides has increased public concern regarding the risks associated with hazardous residues in agricultural products and their adverse effects on ecosystem biodiversity (Meena et al., 2020). In addition, disease control using chemical pesticides is cost-effective for farmers in developing countries (Wesseling et al., 1997; Bordoh et al., 2020). Therefore, biological control using antagonistic microorganisms has emerged as an environment-friendly alternative for controlling plant diseases (Köhl et al., 2019; De la Lastra et al., 2021).

*Brevibacillus* spp. is one of the PGPR groups used as biofertilizers or biopesticides in different crops and against a variety of soil-borne and foliar pathogens (Nehra et al., 2016). Several studies have been conducted on antibiotic production by antagonistic microbes, such as *Bacillus* sp., *Pseudomonas* sp., and *Streptomyces* sp. (Beneduzi et al., 2012; Ngalimat et al., 2021). *Bacillus* spp. produce spores that can be used to develop an effective microbial biopesticide formulation in the form of a biocontrol agent (BCA) (Bonaterra et al., 2022). Several studies have demonstrated that secondary metabolites produced by antagonistic bacteria play key roles in controlling various phytopathogens (Han et al., 2015). In several cases, the control efficacy achieved by *Bacillus* strains has been associated with the production of antifungal and volatile organic compounds (VOCs) that induce plant defense reactions (Khalaf and Raizada, 2018). Whole-genome analysis of *Bacillus* species constitutes the basis for understanding the interactions between plants and other microorganisms (Magno-Perez-Bryan et al., 2015; Shaligram et al., 2016). Using genome mining, many antimicrobial compounds were discovered, such as brevicidine and antimicrobial cyclic lipopeptides, which are found in *Brevibacillus laterosporus* DSM 25 (Li Y. X. et al., 2018). Complete genome sequencing technology has good application prospects for the discovery of genome sequence information of unknown bacteria and the exploration of critical functional genes (Wang et al., 2020).

Considering these potential benefits, this study aimed to (i) screen *in vitro* antagonistic activity of various bacterial isolates obtained from the Freshwater Bioresources Culture Collection (FBCC), Korea, and identify effective antagonistic bacteria with inhibitory effects against major phytopathogens; (ii) evaluate the potential efficiency of the selected PGPR strain B-4359 as a BCA against red pepper anthracnose caused by *C. acutatum* under *in vitro* and field conditions and for plant growth promotion; and (iii) evaluate complete genome sequence of B-4359 and identify the divergent genomic characteristics among other *Brevibacillus* strains for comparative genome analysis.

## Materials and methods

### Antagonistic bacterium and pathogens

All fungal pathogens tested in our study, including *C. acutatum* KACC 42403, *Fusarium oxysporum* f. sp. *lycopersici* KACC 40043, *Colletotrichum coccodes* KACC 48737, and *Colletotrichum fructicola* GYUN-11115 were procured from the Korean Agricultural Culture Collection (KACC), Korea. The antagonistic bacterium *Brevibacillus halotolerans* B-4359 was obtained from the FBCC, Korea. This bacterium was selected based on its antagonistic effects on various bacterial and fungal plant pathogens (provided in detail in the Supplementary material).

### Antagonistic effect of *Brevibacillus halotolerans* B-4359 on solid medium

*Brevibacillus halotolerans* B-4359 was tested for *in vitro* antagonistic activity against five fungal pathogens, including *C. acutatum* KACC 42403, *F. oxysporum* f. sp. *lycopersici* KACC 40043, *C. coccodes* KACC 48737, and *C. fructicola* GYUN-11115, using a dual culture assay. Fungal pathogens were cultured onto potato dextrose agar (PDA) plates (90 mm diameter) at 25°C for 7 days. The bacteria B-4359 was cultured in tryptic soy agar (TSA) plates at 28°C for 2 d. A mycelial plug was removed from the fully grown PDA plate using a sterile cork borer (5 mm in diameter) and inoculated onto PDA medium supplemented with peptone (PDK) on one side 2 cm away from the edge, whereas the bacterium was streaked on the other side. Plates inoculated with fungal disks alone were used as controls. All the plates were incubated at 25°C for 10 d. The distance between B-4359 cells and the mycelium was measured as the inhibition of mycelial growth. The results are expressed as the percentage inhibition of the growth of fungal mycelia in the presence and absence of bacteria using the following equation (Montealegre et al., 2003).


$$\begin{array}{l} \mathrm{Inhibition of mycelial growth}\;\left(\%\right)\\ \;=\left[\left(1-\mathrm{mycelial growth of treatment}/\mathrm{mycelial growth of control}\right)\right]\\ \;\times 100. \end{array}$$

### *In vitro* antifungal effect of cell-free culture filtrate of B-4359 on agar plates

The bacterial cells of B-4359 were cultured in tryptic soy broth (TSB) at 28°C under shaking conditions at 180 rpm for 24 h. The culture broth was centrifuged at 13,000 × g for 5 min at 4°C. The supernatant was collected and sterilized through a membrane filter (0.02 μm), which was used as a culture filtrate (*CF*). The mycelial plugs (5 mm diameter) of *F. oxysporum* f. sp. *lycopersici* KACC 40043, *C. coccodes* KACC 48737, *C. fructicola* GYUN-11115, and *C. acutatum* KACC 42403 were placed onto the center of PDK plates, and sterile paper discs (6 mm diameter) impregnated with 20 μL of the *CF* were placed onto the same plate at four different sides. Paper discs impregnated with TSB were used as untreated controls. Growth was measured 12 d after incubation at 25°C. Each treatment consisted of three replicates and the experiment was performed at least twice.

### Evaluation of volatile organic compounds from B-4359 on inhibition of fungal pathogens

To determine the volatile organic compounds (VOCs) secreted by *B. halotolerans* B-4359 against the growth of fungal pathogens, exposure to *B. halotolerans* B-4359 VOCs was performed using the sandwich method described by Li N. et al. (2018). *F. oxysporum* f. sp. *lycopersici* KACC 40043, *C. coccodes* KACC 48737, *C. fructicola* GYUN-11115 and *C. acutatum* KACC 42403 were cultured on PDA plates at 25°C for 7 d. The antagonistic bacterium B-4359 was cultured on a TSA plate for 48 h. Mycelial plugs (5 mm diameter) of fungal pathogens were placed on the center of freshly prepared PDA plates, and bacteria were streaked onto TSA plates. Bacteria-inoculated plates were placed on top of PDA plates inoculated with mycelial plugs of the two fungal pathogens. Another set of fungal pathogen plates was sandwiched between TSA plates without streaked bacteria and used as a control group. All plates were sealed with a double layer of parafilm. Colony diameter was measured after incubating the *C. acutatum* KACC 42403 inoculated plates at 28°C for 12 d. The experiment was performed twice in triplicates.

### Morphological characteristics of *Brevibacillus halotolerans* B-4359

For microscopic observation, a bacterial colony was picked with a sterile inoculation loop, placed in sterile distilled water (SDW) on a Formvar-coated copper grid, and stained with 2% uranyl acetate for negative staining. The preparation was examined using transmission electron microscopy (TEM; Hitachi, Japan). For bacterial endospore identification, the bacterium was streaked onto freshly prepared TSA plates and incubated at 28°C for 2 d. The bacterial colony was inoculated onto a glass slide and endospore formation was observed using a light microscope (Olympus BX43, Olympus, Tokyo, Japan).

### Inhibition of spore germination of *Colletotrichum acutatum* by treatment with B-4359 cell suspension

To determine whether B-4359 is involved in inhibiting the conidia germination, the conidia from 7-d-old cultures at 25°C on PDA were harvested with SDW and adjusted to 10^5^ spores/mL using a hemocytometer. The bacterial isolate B-4359 was cultured onto a TSA plate for 48 h at 28°C, and the cell suspensions were prepared in three different concentrations (10^6^, 10^7^, and 10^8^ CFU/mL) in SDW. The conidial germination and appressorium formation of *C. acutatum* were tested on a covered glass surface treated with B-4359 bacterial suspensions using a previously described method (Kwak et al., 2012). Briefly, the conidial suspensions (10 μL) were mixed with bacterial suspensions (10 μL) in different concentrations and placed on a glass slide. Conidia suspensions without treating bacterial suspensions were considered as a control. The conidial germination and formation of appressorium and primary hyphae in the B-4359 treatment were assessed during incubation at 25°C and at different durations (0, 6, 18, 24, and 48 h) in Petri dishes containing moist paper. The glass slides were observed under a light microscope (Olympus BX43, Olympus, Tokyo, Japan). All the experiments were performed thrice in triplicate. The conidial germination inhibition rate (%) was calculated using the following formula:


$$\begin{array}{l} \mathrm{Conidial germination inhibition rate}\;\left(\%\right)\\ =\left(\mathrm{germination of treated conidia}/\mathrm{germination of control}\right)\times 100. \end{array}$$

### Effect of B-4359 treatment on *in vitro* disease suppression of anthracnose

To investigate the effect of B-4359 on anthracnose disease suppression in red peppers, fresh and healthy red pepper fruits of similar sizes were surface-sterilized in 1% NaOCl for 1 min and rinsed twice with SDW. The fungal pathogen *C. acutatum* GYUN-10586 causes anthracnose on red pepper and was isolated from the infected red pepper field, cultured onto PDA plates at 25°C, and used in our study for inoculation. Five wounds were made on one red pepper fruit using a sterile needle and B-4359 suspensions (10 μL) at 10^6^ and 10^7^ CFU/mL were dropped on each wound, allowed to dry, and the fruits were inoculated with 10 μL of fungal pathogen spore suspension (10^5^ spore/mL) on each wound just by dropping. Pepper fruits treated with SDW served as a control. All fruits were placed on square plates (40 cm × 40 cm) containing moist paper. The disease severity (%) was recorded based on disease rating after incubating the plates at 28°C for 5 d. The disease severity (%) was calculated as follows: Based on the score, a disease index scale was established from 0 to 5 (0 = no symptoms, 1 = lesions with <10% disease incidence, 2 = symptoms with 11–20%, 3 = 21–40%, 4 = 41–70%, and 5 = symptoms with >71–100%. Disease severity (%) = [sum (class frequency × score of rating class)]/[(total number of fruits) × (maximal disease index)] × 100). The experiment was performed twice with five replicates.

### Plant growth-promotion effect of B-4359 cell suspensions on red-pepper

In order to confirm the plant growth-promoting ability of B-4359, 3-week-old red-pepper seedlings were sown in plastic pots (10 cm diameter) containing horticultural soil (Baroker, Seoul Bio Co., Ltd., Eumseong, Korea). All the pots were treated with B-4359 bacterial suspensions at various concentrations (10^5^, 10^6^, and 10^7^ CFU/mL) by soil drench (SD). Red pepper seedlings treated with SDW served as the control group. The plant growth (height) was measured after maintaining the plants at 25°C and 12 h/12 h (light/dark) for 20 d in a plant growth chamber.

### Assessment of growth curves and indole-3-acetic acid production

To determine the growth curve of B-4359, a single bacterial colony of B-4359 cultured on TSA plates for 24 h was inoculated in TSB and incubated at 28°C for 72 h under shaking conditions (180 rpm). The optical density (OD) of culture broth was measured at 600 nm at 4 h intervals using a spectrophotometer, and 10 μL of the culture was inoculated onto TSA plates to check the number of colonies, from which CFU/mL value was calculated. The ability of B-4359 to produce IAA was also investigated. B-4359 was incubated at 180 rpm 28°C in TSB with tryptophan (500 μg/mL). The bacterial culture was centrifuged at 10,000 × *g* for 15 min, and 1 mL supernatant was transferred to a fresh tube to which 50 μL of 10 mM orthophosphoric acid and 2 mL of Salkowski reagent (1 mL of 0.5 M FeCl_3_ in 50 mL of 35% HClO_4_) were added. The mixture was incubated at room temperature (25°C) for 25 min and the absorbance of the pink color that developed was measured at 530 nm. The standard IAA solutions of 5, 10, 20, 50, and 100 μg/mL were prepared with culture medium, respectively. Absorbance was measured at 530 nm to obtain a standard curve.

### Field evaluation of B-4359 treatment against red-pepper anthracnose

To determine the effect of antagonistic B-4359 bacteria against red pepper anthracnose disease under field conditions, two-month-old healthy red pepper (cv. color king) seedlings were purchased from a local seedling market and transplanted into the field at a spacing of 40 × 100 cm. The experiment was conducted in a field at Andong National University, South Korea. The treatments were grouped as follows: control, chemical control, pyraclostrobin, pre-immersion with B-4359 (PI B-4359), foliar spray (FS) with B-4359 (FS B-4359), and foliar spray + soil drench with B-4359 (FS + SD B-4359). In the PI with B-4359 cell suspensions, the roots were pre-immersed in bacterial suspensions of B-4359 at 10^6^ CFU/mL for 2 h before they were transplanted into the field. For the foliar spray treatment group, 250 mL/plant was used, whereas 500 mL/plant was used for the soil drench treatment group. After transplantation, all treatments were administered six times (10 d intervals) during the growth period of 60 d; the detailed treatment schedule is provided in Supplementary Table 1. Two months after transplantation, the first treatment was applied to red pepper plants. The disease rate (%) of anthracnose was calculated from disease-infected red pepper fruits after harvesting 2 weeks after the last treatment under field conditions. The disease rate (%) was calculated using the following formula: Disease rate (%) = Number of diseased fruits/total number of fruits × 100.

### Induced resistance genes in B-4359 treated red peppers

To investigate the effect of B-4359 on the expression of defense genes in red pepper plants, 2-month-old red pepper seedlings were treated with 50 mL of B-4359 cell suspensions (10^6^ CFU/mL), 1 mM BTH (positive control) or SDW (negative control) using the foliar spray method. The leaves were sampled at 6 h and 24 h after treatment and stored at −80°C. Total RNA was isolated from red pepper leaf tissues using a RNeasy Plant Mini Kit (Qiagen, Hilden, Germany), following the manufacturer’s instructions. The cDNA synthesis was performed using iScript cDNA Synthesis Kit (Bio-Rad); real-time PCR was performed using SsoAdvanced Universal SYBR Green supermix (Bio-Rad) with housekeeping genes ACT F (5’-TGTTATGGTAGGGATGGGTC-3′), ACT R (5’-TTCTCTCTATTTGCCTTGGG-3′) (Wan et al., 2011), and pathogenesis-related genes CaPR1 F (5’-ACTTGCAATTATGATCC ACC-3′), CaPR1 R (5’-ACTCCAGTTACTGCACCATT-3′) (Yang et al., 2009). The thermocycler parameters were as follows: initial polymerase activation, 10 min at 95°C, and then 40 cycles of 30 s at 95°C, 60 s at 55°C and 30 s at 72°C. The expression level of the *CaPR1* gene was compared by calculating relative transcript quantification using the ΔΔCT method.

### Whole genome sequencing, COG annotation, and secondary metabolites of B-4359

Bacterial genomic DNA was extracted using a Solg Genomic DNA Prep Kit (Solgent, Daejeon, South Korea), following the manufacturer’s instructions. The concentration of the extracted DNA was determined using a Qubit 2.0 fluorometer (Invitrogen, Carlsbad, CA, United States). DNA contamination of the cultured strain was tested by sequencing the 16S rRNA gene using an ABI 3730 DNA sequencing machine (Applied Biosystems, Foster City, CA, USA). The integrity of the gDNA was verified using agarose gel electrophoresis and quantified using a Qubit 2.0 fluorometer (Invitrogen). Sequencing libraries were prepared according to the manufacturer’s instructions for 20 kb template preparation using the BluePippin Size-Selection System and PacBio DNA Template Prep Kit 1.0. Briefly, 10 μg of the gDNA was sheared to 20 kb using g-tubes (Covaris, Woburn, MA, United States); they were then purified, end-repaired, and the blunt-end SMARTbell adapters were ligated. The libraries were quantified using a Qubit 2.0 fluorometer (Invitrogen) and qualified using a DNA 12,000 chip (Agilent Technologies, Waldbronn, Germany). Subsequently, the libraries were sequenced using PacBio P6C4 chemistry in an 8-well SMART Cell v3 at PacBio RSII.

### Secondary metabolites from *Brevibacillus halotolerans* B-4359

Five genes were selected based on the results of secondary metabolite analysis using antiSMASH. The PCR primers for the target genes from B-4359 and the housekeeping gene (16S rRNA) are shown in Table 1. Each primer name represents a gene corresponding to brevicidine for bre, macrobrevin for MAC, petrobactin for ptr6, basiliskamide for bask, bogorol A for bogA, and *Brevibacillus* 16S rRNA for br16. Total RNA from B-4359 cells was obtained from one-day-old cultures grown on TSA using the RNeasy Plus Mini kit (Qiagen, Valencia, CA, United States) according to the manufacturer’s instructions. The cDNA synthesis in 20 μL was performed using an iScript cDNA Synthesis Kit (Bio-Rad) following the manufacturer’s instructions. The SsoAdvanced Universal SYBR Green SuperMix (Bio-Rad) was used for real-time PCR analysis. Real-time PCR mixture in 10 μL of the total volume contained 5 μL of SYBR Green, 2 μL of each primer (5 pM), 2 μL of cDNA, and 1 μL of nuclease-free water. The thermal cycling program was set at 95°C for 3 min, 40 cycles at 95°C for 10 s, and 55°C for 30 s, followed by 55–95°C melting curve analysis.

**Table 1:** List of real-time PCR primers used for the target genes of secondary metabolites.

Primer name	DNA sequence (5′-3′)	Product size (bp)	Source
bre	F GGTGGGTTCCAAAATGTGCC	200	This study
	R CCACTCGCAAAAACCTGACG		
MAC1	F CGAGTCCCATGCTTTGCCTA	200	This study
	R TCAAGGTCACACACATCCCG		
ptr6	F ATCCATCGAAACGGGGTTCC	200	This study
	R CTCGTACAAGCGAGGGATCG		
bask	F CCTTGCATTGTGAACACCCG	200	This study
	R GGTACTCGATGTGTTCGGGG		
bogA1	F GTGGCTCGAAATGACGAGGA	200	This study
	R ATCAGGCACTGGTAACGCTC		
Br16	F AACGATGAAGGCTTTCGGGT	200	This study
	R AGACTTACATAGCCACCTGCG		

### Additional experiments

Additional experiments, including a collection of fungal and bacterial pathogens, *in vitro* screening of antagonistic bacteria, *in vitro* enzyme activities by antagonistic bacteria, phosphate solubilization assays, siderophore production assays, and auxin and gibberellin detection assays, are described in detail in the Supplementary material.

### Statistical analysis

Data were subjected to analysis of variance (ANOVA) using SAS JMP software (SAS Institute, Cary, NC, United States; SAS, 1995). Significant differences between treatment means were determined using the least significant difference (LSD) at *p* < 0.05. All the experiments were performed at least twice. For each experiment, data were analyzed separately. The results of a representative experiment are presented herein.

## Results

### *In vitro* screening of antagonistic bacteria against phytopathogens

A total of 856 bacterial isolates were obtained from the FBCC, of which 57% were isolated from freshwater, 39% from the soil, and the remainder from sludge and plants. All the isolates were classified into 442 species and 182 genera (Table 2). Most of these genera come under the phylum Pseudomonadota, which accounts for the largest proportion of freshwater, soil, and other sources; while in the sludge, the phylum Bacillota was the most abundant (Supplementary Figure 1A). Among the strains isolated from freshwater, *Flavobacterium* sp. was the most common (9%), followed by *Pseudomonas* sp. and *Rhodoferax* sp., which accounted for 5% of the freshwater isolates. Of the strains isolated from the sludge, 50% were *Bacillus* spp., from which 20% were from the soil (Supplementary Figure 1B). Among all the isolates used for *in vitro* screening, only 65 strains exhibited antagonistic activity against at least one of the five pathogens used in our study, with a hit rate of 7.6%. Furthermore, the 65 strains were classified into 43 species and 25 genera. Of the isolates obtained from soil, 87% had no antagonistic ability, *Bacillus* sp., which accounted for 4%, and *Pseudomonas* sp., which accounted for 3%, showed the greatest antagonistic effect. In addition, 96% of strains from freshwater had no antagonistic ability and approximately 1% of *Lactobacillus* spp. showed the greatest antagonistic effect, and the other antagonists were less than 1% (data not shown).

**Table 2:** Number of strains, species, and genera of the screened bacterial isolates used in this experiment.

	No. of		
	Strains	Species	Genera
Total number of strains used in the experiment	856	442	182
Number of strains antagonizing at least one pathogen	65	43	25
Ratio of antagonistic bacteria (%)	7.6	9.7	13.7

As observed from the antifungal activity test, 44 strains inhibited the growth of the fungal pathogen *C. acutatum* with a hit rate of 5%; 15 strains inhibited the growth of the fungal pathogen *F. oxysporum* with a hit rate of 1.8% (Table 3). Antagonistic bacterial species, such as *Streptomyces* and *Brevibacillus*, were found to exhibit greater antifungal activity than other species in our study against *C. acutatum* and *F. oxysporum* (Supplementary Table 2). The strain *Streptomyces sporoverrucosus* B-1662 exhibited the greatest antagonistic activity against *C. acutatum* with an average inhibition percentage of 81, whereas the growth of the fungal pathogen *F. oxysporum* was strongly inhibited by the antagonistic strain *S. virginiae* B-4370, with an inhibition percentage of 71.1% (Supplementary Table 2). Three isolates, *S. virginiae* B-4371, *Brevibacillus halotolerans* B-4366, and *S. sporoverrucosus* B-1662, showed greater antagonistic activity against *F. oxysporum* with inhibition percentages of 68.3, 68.3, and 63.2, respectively (Supplementary Table 2).

**Table 3:** List of the five phytopathogens tested in this study and their culture conditions, and a summary of the antagonistic activity results of all 856 isolates against pathogens.

Pathogen		Host	Culture Condition (Medium, Temperature)	Source	No. of antagonistic bacteria	Ratio of antagonistic bacteria (%)
Fungi	*Colletotrichum acutatum* 42,403	Pepper	PDA, 25°C	KACC	44	5.0
	*Fusarium oxysporum* f. sp. *lycopersici* 40,043	Tomato	PDA, 25°C	KACC	15	1.8
Bacteria	*Xanthomonas arboricola* pv*. Juglandis* GYUN-39	Walnut fruit	TSA, 28°C	Lab strain	11	1.3
	*Pectobacterium carotovorum* GYUN-18	Tomato stem	TSA, 28°C	Lab strain	13	1.5
	*Erwinia pyrifoliae* GYUN-550	Apple leaf	TSA, 28°C	Lab strain	6	0.7

In the case of the antibacterial activity test, 11, 13, and 6 strains showed antagonistic activity against the bacterial pathogen, *Xanthomonas arboricola* pv*. juglandis* GYUN-39; *Pectobacterium carotovorum* GYUN-18; and *Erwinia pyrifoliae* GYUN-550. The hit rate was determined to be 1.3, 1.5, and 0.7% for *X. arboricola*, *P. carotovorum*, and *E. pyrifoliae*, respectively (Table 3). Some species such as *Paenibacillus terrae* and *Bacillus mycoides*, exhibit antagonistic activity against *X. arboricola*. The three isolates of *P. terrae* showed different antagonistic activities against the three pathogenic bacteria. Among them, the isolate B-248 exhibited an antagonistic effect on *C. acutatum, F. oxysporum, E. pyrifoliae*, and *X. arboricola* but no activity against *P. carotovorum* (Supplementary Table 3). Two antagonistic strains, such as *Lactobacillus plantarum* subsp. *argentoratensis* and *B. halotolerans* exhibited greater antagonistic activity against *P. carotovorum* than the other strains. These three isolates were obtained from freshwater. In contrast, the other two strains, B-367 and 368, which were also isolated from the same area, showed no antagonistic effect against any of the five pathogens (data not shown). Similarly, antagonistic strains such as *B. halotolerans* and *Chromobacterium alkanivorans* exhibited the highest antagonistic activity against the bacterial pathogen *E. pyrifoliae* (Supplementary Table 3). From *in vitro* screening, nine strains exhibiting substantial antagonistic activity (in mm diameter) against phytopathogens were selected for further studies. The strain B-4359 was selected for further studies based on its antagonistic activity against several phytopathogens and various enzyme activities. The strain B-4359 was evaluated for the control of red pepper anthracnose because of the increased incidence of the disease in Korea.

### *In vitro* enzyme activities, siderophore production, phosphate solubilization, indole, and gibberellin production by bacteria

Nine effective strains were evaluated for starch hydrolase, cellulase, protease, and chitinase activities, and each strain displayed different enzyme activities *in vitro* (Supplementary Figure 2). Strain B-4359 produced protease at a greater level and a lower level of chitinase, but two other enzymes, starch hydrolase, and cellulase, were not produced by the bacteria. The inhibition zones around the inoculation sites on the solid media in the Petri dishes were determined by the degradation of various substrates, such as soluble starch, CMS, proteins, and chitin, by amylase, cellulase, protease, and chitinase, respectively. In contrast, bacterium B-4359 is involved in the production of either siderophores or phosphate solubility (Supplementary Figure 2). This indicates that the mode of action of the bacterium might involve other mechanisms. Indole and gibberellin content were analyzed from our bacteria isolate B-4359, but it could not produce indole; while gibberellin was produced at 855.6 μg/mL (Supplementary Figure 3). This indicates that the strain may promote plant growth.

### Morphological characteristics of B-4359

**Figure 1:**
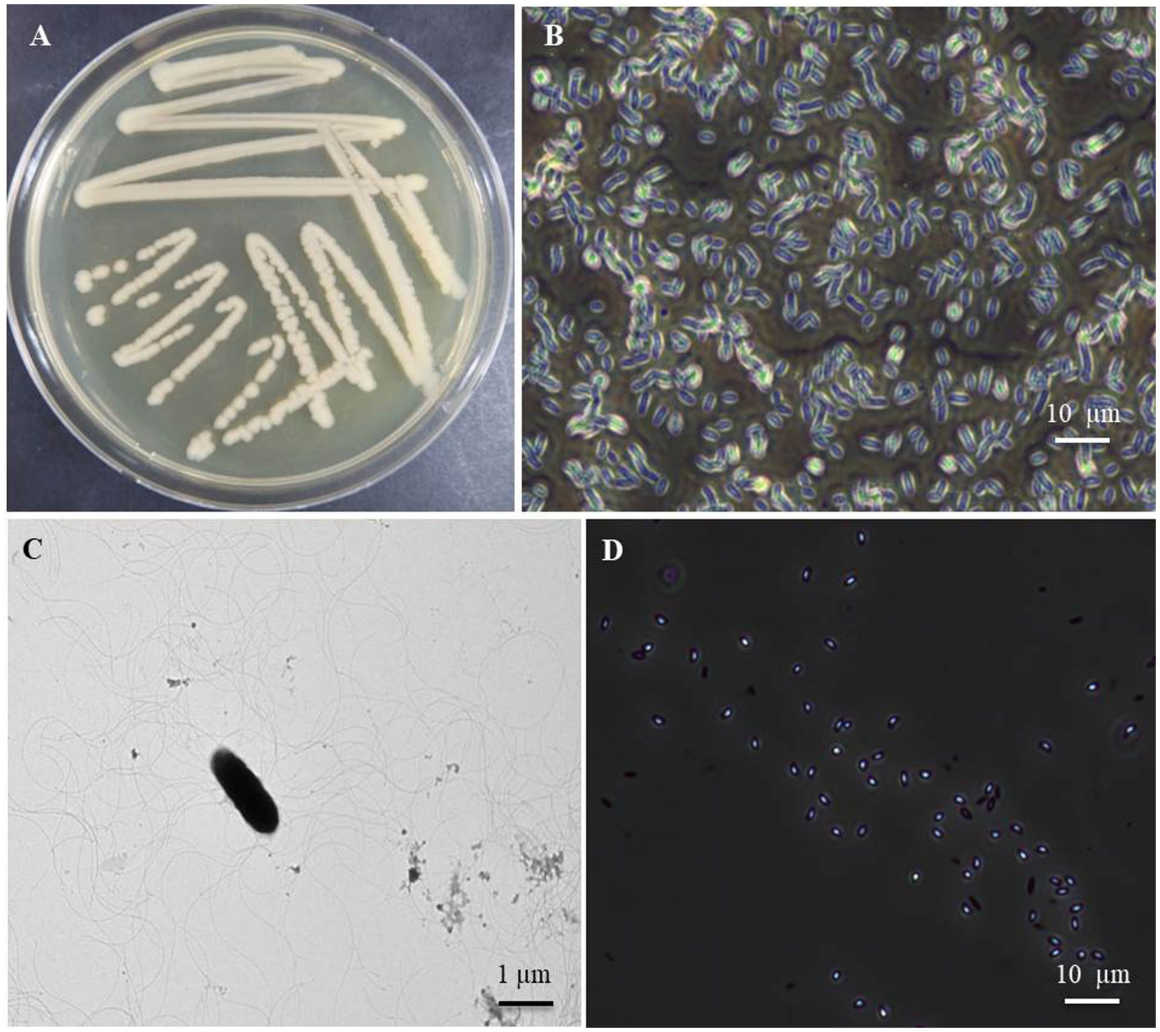
Cultural and morphological characteristics of the bacterial isolate *B. halotolerans* B-4359 using transmission electron microscopy (TEM) and light microscope. **(A)** Bacteria cultured at 28°C for 48 h showed smooth glossy and rod-shaped colonies, **(B)** Bacterial cells appeared rod-shaped under light microscopy with 400X magnification, and **(C)** rod-shaped bacteria appears with peritrichous flagella through TEM (Bar = 1 μm). Bar = 10 μm. **(D)** Endospores were observed in the 7-day-old cultured broth (Bar = 10 μm).

Morphological characteristics of the *B. halotolerans* isolate B-4359 obtained from FBCC were smooth, glossy, light brown, and flat colonies when cultured on TSA medium after 48 h incubation at 28°C (Figure 1A). When observed using a TEM, the bacterial cells were found to be rod-shaped, measuring approximately 0.84–1.49 μm × 2.08–4.10 μm (width × length; Figure 1B), motile possessing peritrichous flagella (Figure 1C). The bacterial cells were found to be gram-positive using Gram staining, and endospores were observed when the bacteria were cultured for 7 d in TSA (Figure 1D). Most of these characteristics are similar to those of *Brevibacillus halotolerans* sp. nov., as described by Song et al. (2017).

### *In vitro* antagonistic ability of B-4359 against fungal phytopathogens

**Figure 2:**
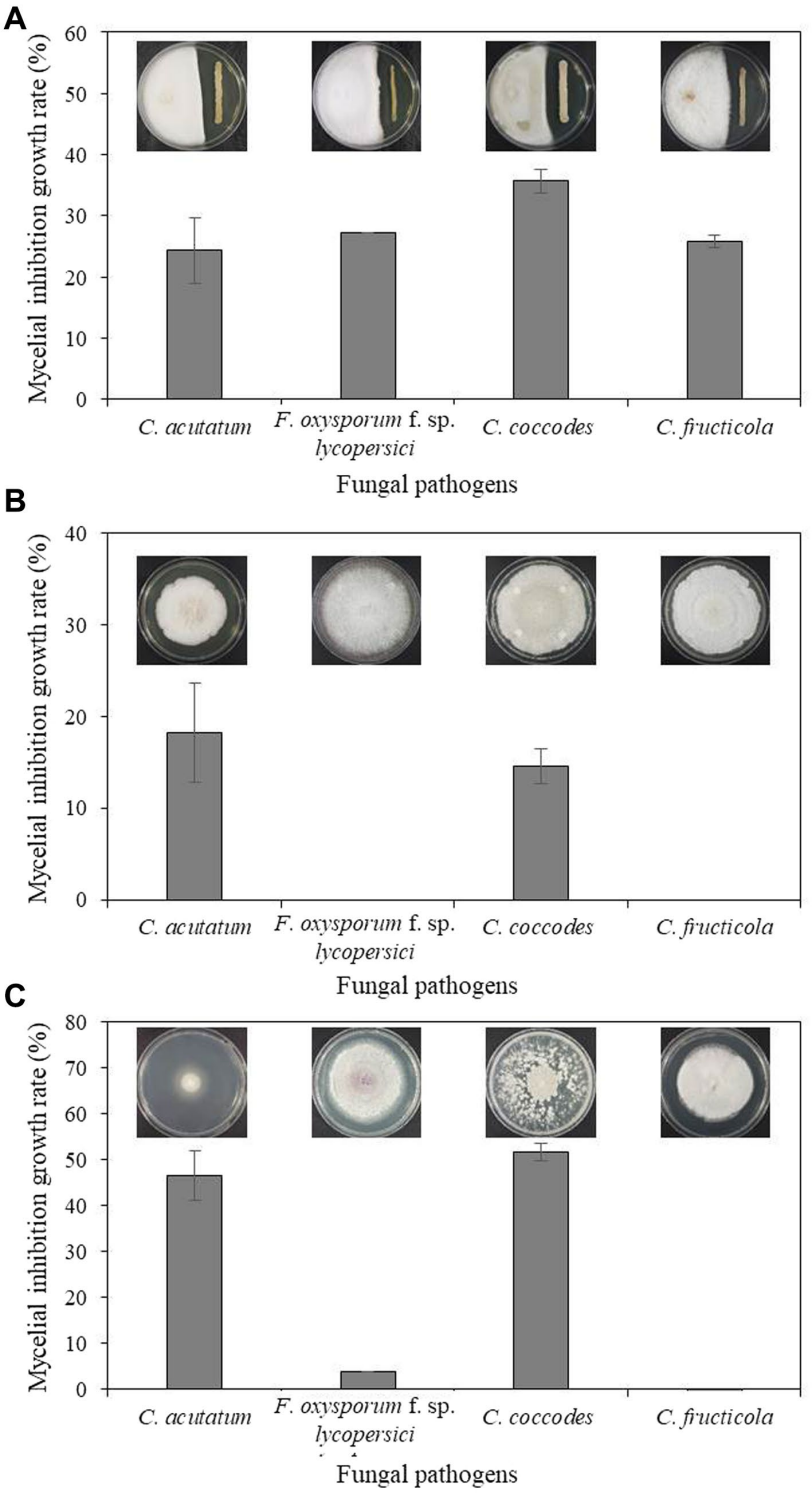
**(A)** *In vitro* antagonistic effect *of B. halotolerans* B-4359 against fungal plant pathogens. An *in vitro* antagonistic activity of *B. halotolerans* B-4359 against fungal pathogens (*C. acutatum*, *F. oxysporum* f. sp. *lycopersici*, *C. coccodes*, and *C. fructicola*) was tested using a confrontation plate assay by inoculating the mycelial plug at 2.0 cm away from one edge of the PDK plates and streaked with bacterial cultures at another edge on the same plate. The incubation days were 13, 10, 10, and 7 days for *C. acutatum*, *F. oxysporum*, f. sp. *lycopersici*, *C. coccodes*, and *C. fructicola*, respectively. **(B)** Inhibitory effect of culture filtrate (*CF*) of antagonistic bacterium *B. halotolerans* B-4359 on the fungal growth of *C. acutatum*. Paper discs impregnated with TSB were used as a non-treated control. The diameter of mycelial growth of fungal pathogens on PDK plates was recorded 12 d after incubation at 25°C. **(C)** The inhibitory effect of volatile organic compounds (VOCs) produced by *B. halotolerans* B-4359 against the growth of fungal pathogens was tested using a sandwich method. The experiment was performed twice with triplicates.

Bacterium B-4357 was tested for *in vitro* antagonistic activity against four fungal pathogens, including *C. acutatum* KACC 42403, *F. oxysporum* KACC 40043, *C. coccodes* KACC 48737, and *C. fructicola* GYUN-11115, which cause anthracnose, Fusarium wilt, leaf anthracnose, and apple bitter rot, respectively. Mycelial growth of all pathogenic fungi was inhibited by the B-4359 suspensions to a greater extent than that of the untreated control (Figure 2A). Among the four fungal pathogens, mycelial growth of *C. coccodes* KACC 48737 was greatly inhibited by B-4359 13 d after incubation at 25°C, when compared with the growth inhibition of other three pathogens. B-4359 showed inhibition percentages of 24.3, 27.2, 35.7, and 27.7 for *C. acutatum* KACC 42403, *F. oxysporum* KACC 40043, *C. coccodes* KACC 48737, and *C. fructicola* GYUN-11115, respectively.

### *In vitro* mycelial growth inhibition effect *CF* and VOCs produced by B-4359

The cell-free *CF* of strain B-4359 was tested for the growth of *C. acutatum*. The colony diameter of the B-4359-*CF*-treated fungi was reduced relative to that of the untreated control (Figure 2B). The resulting inhibition rates of mycelial growth were 18.21 and 14.63% for *C. acutatum* KACC 42403 and *C. coccodes* KACC 48737, respectively, in the B-4359-*CF* treatment. This indicated that the presence of secondary metabolites in our antagonistic bacteria played a role in the growth inhibition of fungal pathogens. Furthermore, we tested whether VOCs produced by the antagonistic bacterium *B. halotolerans* B-4359 inhibited the growth of *C. acutatum* KACC 42403 *in vitro* (Figure 2C). The pathogen *C. acutatum* was inhibited by the VOCs produced by *B. halotolerans* B-4359 to a greater level using the sandwich method. The mycelial growth inhibition rates of *C. acutatum* KACC 42403 and *C. coccodes* KACC 48737 were 46.3 and 51.7%, respectively, by treatment with the presence of VOCs released from *B. halotolerans* isolate B-4359. This resulted in strain B-4359 having the ability to produce VOCs against the growth of *C. acutaum*. The mycelial growth of *F. oxysporum* KACC 40043 and *C. fructicola* GYUN-11115 was not inhibited by the VOCs produced by the B-4359 strain.

### Effect of B-4359 cell suspensions on *Colletotrichum acutatum* spore germination

**Figure 3:**
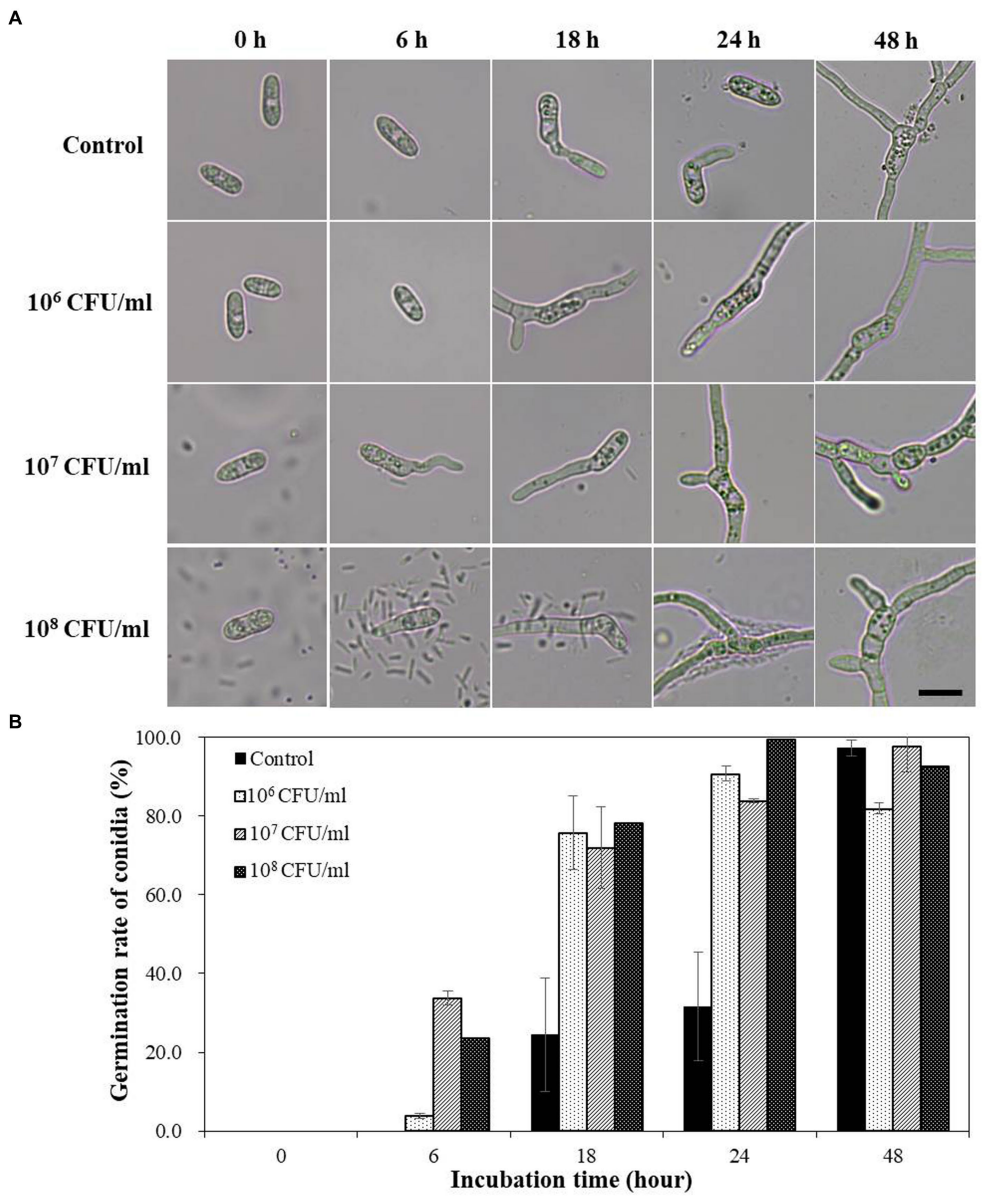
Effect of B-4359 cell suspensions on conidial germination rate and microscopic observation. **(A)** Microscopic observations of the fungal spore germination of *C. acutatum* GYUN-10586 after *B. halotolerans* B-4359 treatment during the incubation period from 0 to 48 h. (Bar = 10 μm). **(B)** The percentage of conidial germination rate by treatment with B-4359 cell suspensions was compared to the non-treated control. The experiment was repeated three times with three replicates per treatment producing similar results. The representative image is presented here.

When the conidial spores of *C. acutatum* were treated with B-4359 bacterial suspensions in various concentrations under *in vitro* conditions, spore germination occurred faster than in the non-treated control (Figure 3A). No marked difference was observed in appressorium formation between the B-4359 treatment and control groups. B-4359-treated conidia at 10^7^ CFU/mL resting on the hard glass surface started to germinate after the 6^th^ hour of incubation, whereas water-treated conidia did not germinate. Similarly, the spore germination rate (%) had not exceeded 30% after the 18th h of incubation, whereas B-4359-treated conidia showed a germination rate of more than 70% at all concentrations (Figure 3B). These results suggest that B-4359 cell suspensions do not play a role in suppressing the growth of conidial spores of the fungal pathogen *C. acutatum in vitro*.

### Control of red-pepper anthracnose by B-4359 suspensions

**Figure 4:**
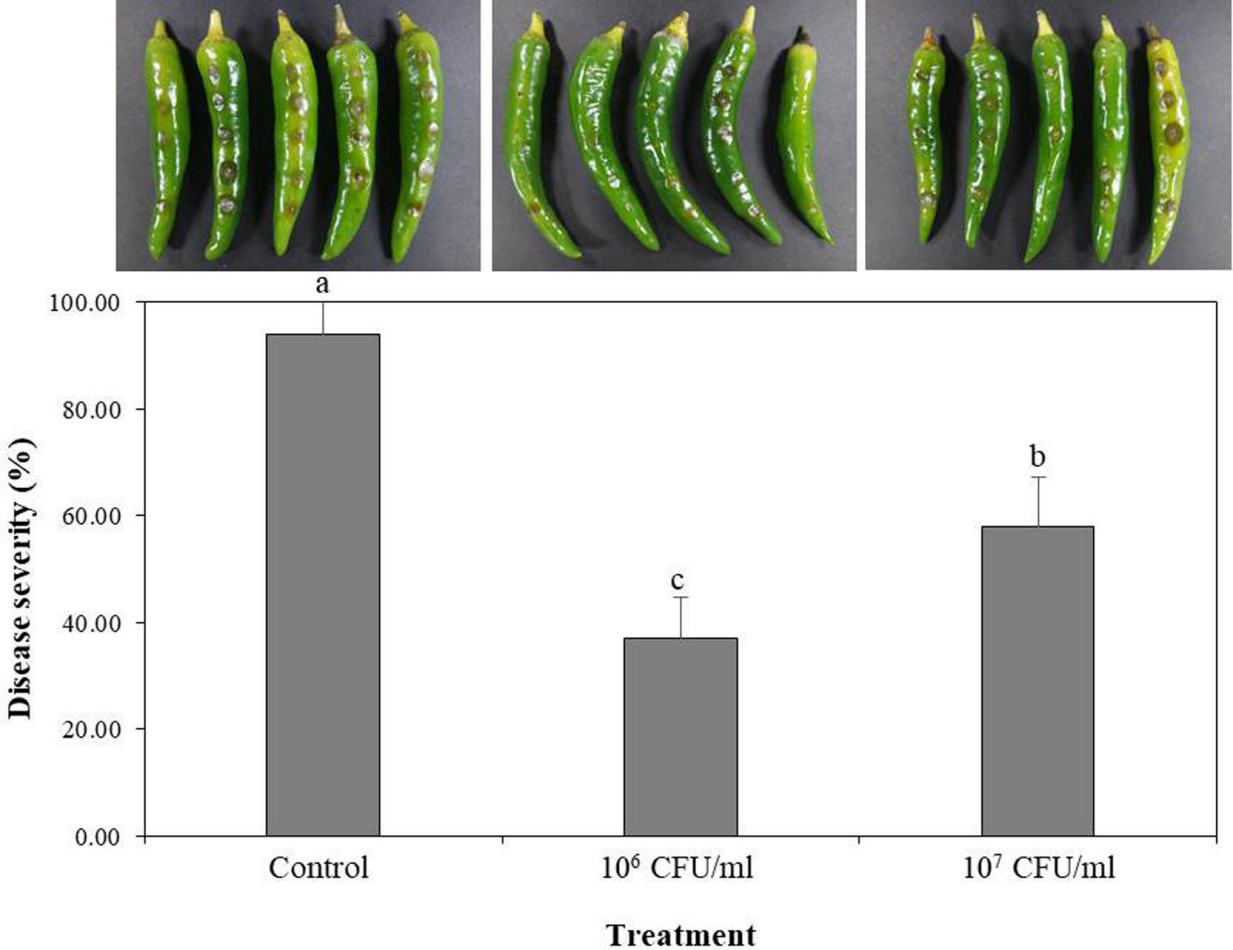
Effect of *B. halotolerans* B-4359 cell suspensions on suppression of disease severity (%) of anthracnose caused by *C. acutatum* GYUN-10586 on red-pepper fruits. Red-pepper fruits treated with SDW served as a control. The experiment was performed two times with five replicates. The results were compared with a non-treated control 3 days after incubation at 25°C. The experiment was performed two times with three replicates. Bars with the same letters do not differ from each other according to the least significant difference (LSD) (*p* < 0.05).

B-4359 was tested for its preventive effect against the anthracnose pathogen *C. acutatum* GYUN-10586, a laboratory-owned strain isolated from our red pepper field at Andong National University. Disease severity (%) was reduced to a greater level at both concentrations of B-4359 suspensions in comparison with a non-treated control after 5 days of incubation (Figure 4). Disease severity was 37 and 58% in B-4359-treated plants at 10^6^ and 10^7^ CFU/mL, respectively, whereas it was 94% in non-treated control.

### Bacterial growth curve of B-4359 and production of IAA

**Figure 5:**
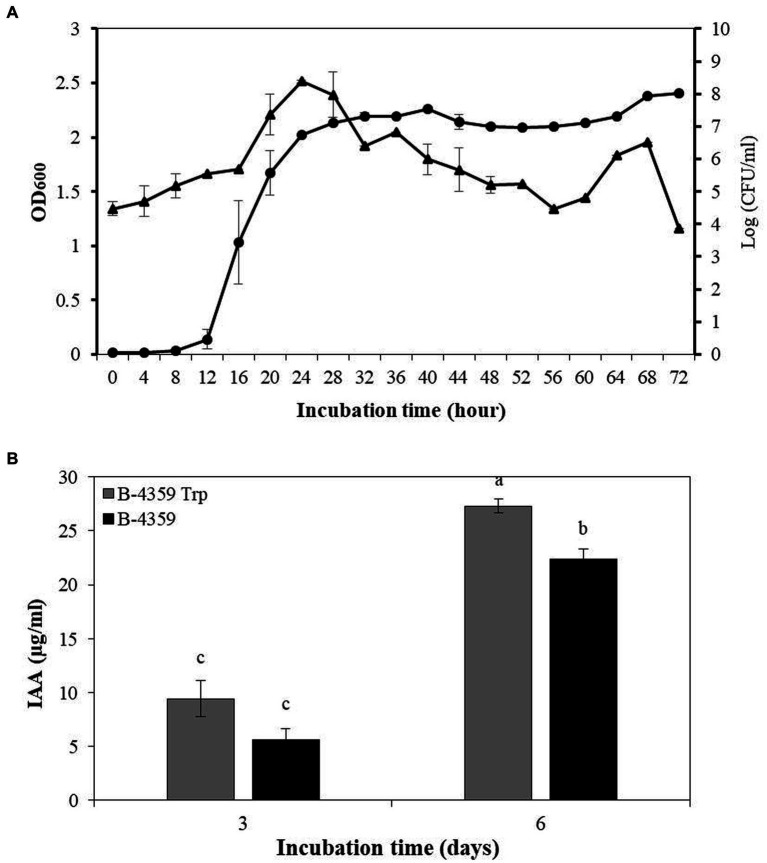
Bacterial growth curve and IAA production of *B. halotolerans* B-4359. **(A)** Bacterial growth curve of *B. halotolerans* B-4359 incubated in TSB at 28°C and 180 rpm. The optical density (OD) was measured at 600 nm in every 4 h interval. The plot represents as OD_600_ (●) and Log (CFU/mL) (▲). **(B)** IAA production by *B. halotolerans* B-4359. The absorbance of pink color development at 530 nm was measured. B-4359 was incubated in TSB with tryptophan (500 μg/mL) at 28°C and 180 rpm. An IAA standard curve was used to quantify the absorbance of the pink produced at 530 nm.

Bacterial growth curve analysis of B-4359 in liquid culture revealed that the growth has increased rapidly from 12 h onwards. This was determined by measuring the absorbance of the culture broth at an optical density (OD) of 600 nm using a spectrophotometer (Figure 5A). The growth of bacteria remained in the stationary phase from 24 to 72 h. Bacterial colonies were found to be the highest after 24 h of incubation on TSA medium at 10^8^ CFU/mL, and the number of bacterial colonies decreased until 56 h of incubation and thereafter started to increase again. Isolate, B-4359 was further assayed for IAA production in association with plant growth-promoting activity. Strain B-4359 was more effective in IAA detection than the untreated control (Figure 5B). The IAA production detected on the sixth day by B-4359 cells was about 3-fold increased at 27.3 μg/mL, whereas on the third day, IAA was detected at 9.4 μg/mL. This indicated that B-4359 synthesis high amounts of IAA, depending on exogenous tryptophan. IAA detection in B-4359 effectively promoted plant growth.

### Effect of B-4359 on disease control of red-pepper anthracnose in field conditions

**Figure 6:**
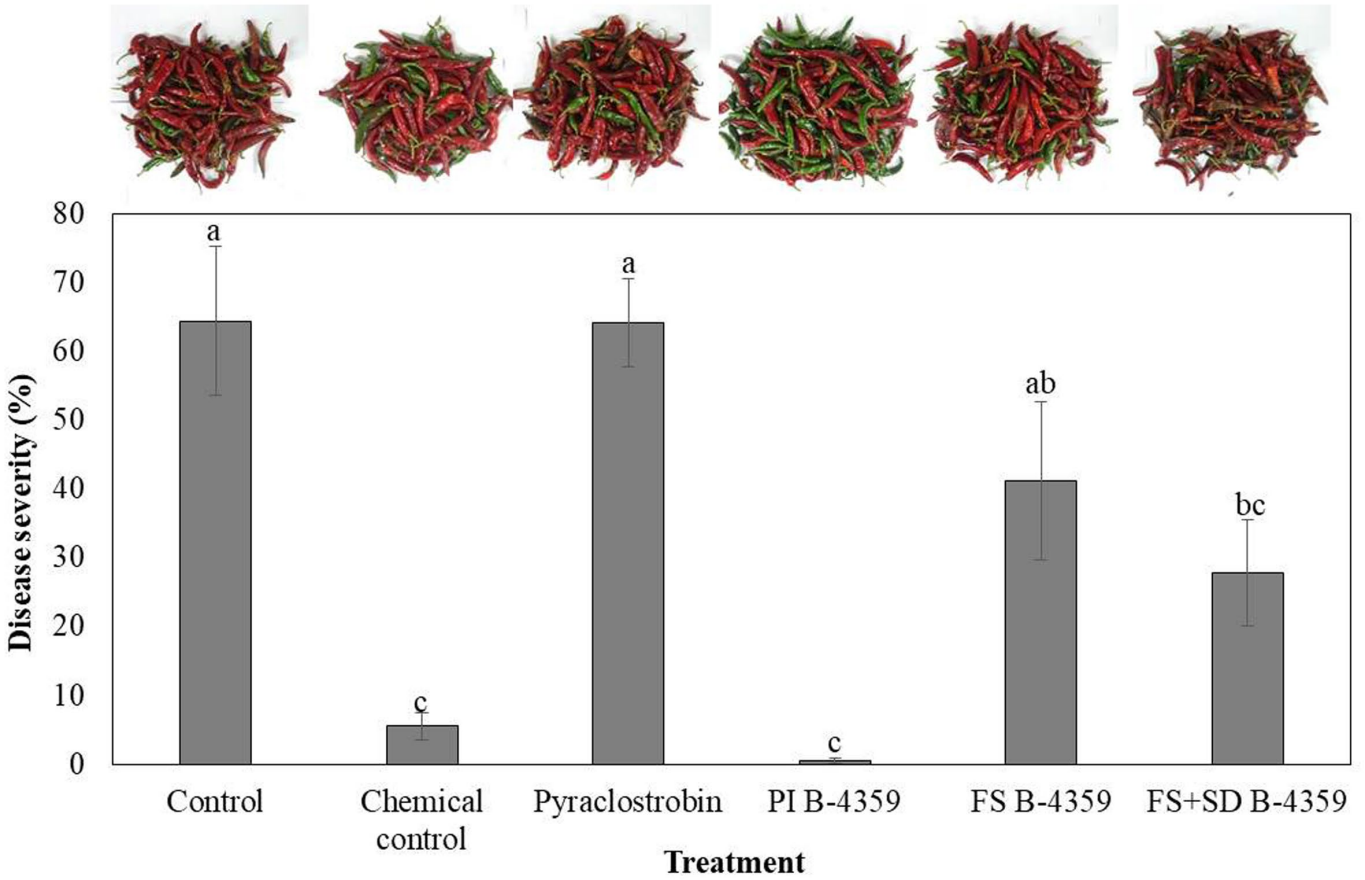
Suppression of anthracnose by treatment with B-4359 under field conditions. After transplanting two-month-old red-pepper seedlings in the field, the plants were treated with B-4359 by soil drench or foliar spray or mixing of both, control (water), chemical control, pyraclostrobin (negative control) six times in 60 d. Disease rate (%) was recorded from disease-infected red-pepper fruits after 10 days of the last treatment. The data for B-4359 were compared with the control. For all the treatments, three different plots with 20 replicates (plants) were used. Differences in letters on bars indicate statistically significant between the treated and the control according to LSD (*p* < 0.05). PI: pre-immersion, FS: foliar spray, SD: soil drench.

Bacterium B-4359 was tested for its ability to suppress red pepper anthracnose caused by *C. acutatum* under field conditions. The B-4359 treatment showed a significant (*p* < 0.05) effect on the reduction of anthracnose, with a disease rate of 0.4% by pre-immersion, compared with the foliar spray or foliar spray + soil drench, with 41.1 and 27.8%, respectively, 2 weeks after the last treatment. When red pepper seedlings were treated with B-4359 by pre-immersion for the first time, followed by chemical treatments exhibited a greater reduction of anthracnose disease than when plants were treated with various chemical fungicides at all six times without any pre-immersion. Overall, the treatment with B-4359 by foliar spray or foliar spray + soil drench showed better performance in controlling disease incidence (%) than the chemical treatment (pyraclostrobin) and a non-treated control (Figure 6). For all treatments, the disease rate (%) in the first experiment was lower than that in the second experiment. This may be due to the development of resistance at an earlier stage of bacterial treatment.

### Whole genome sequencing, COG annotation, and secondary metabolites of B-4359

**Figure 7:**
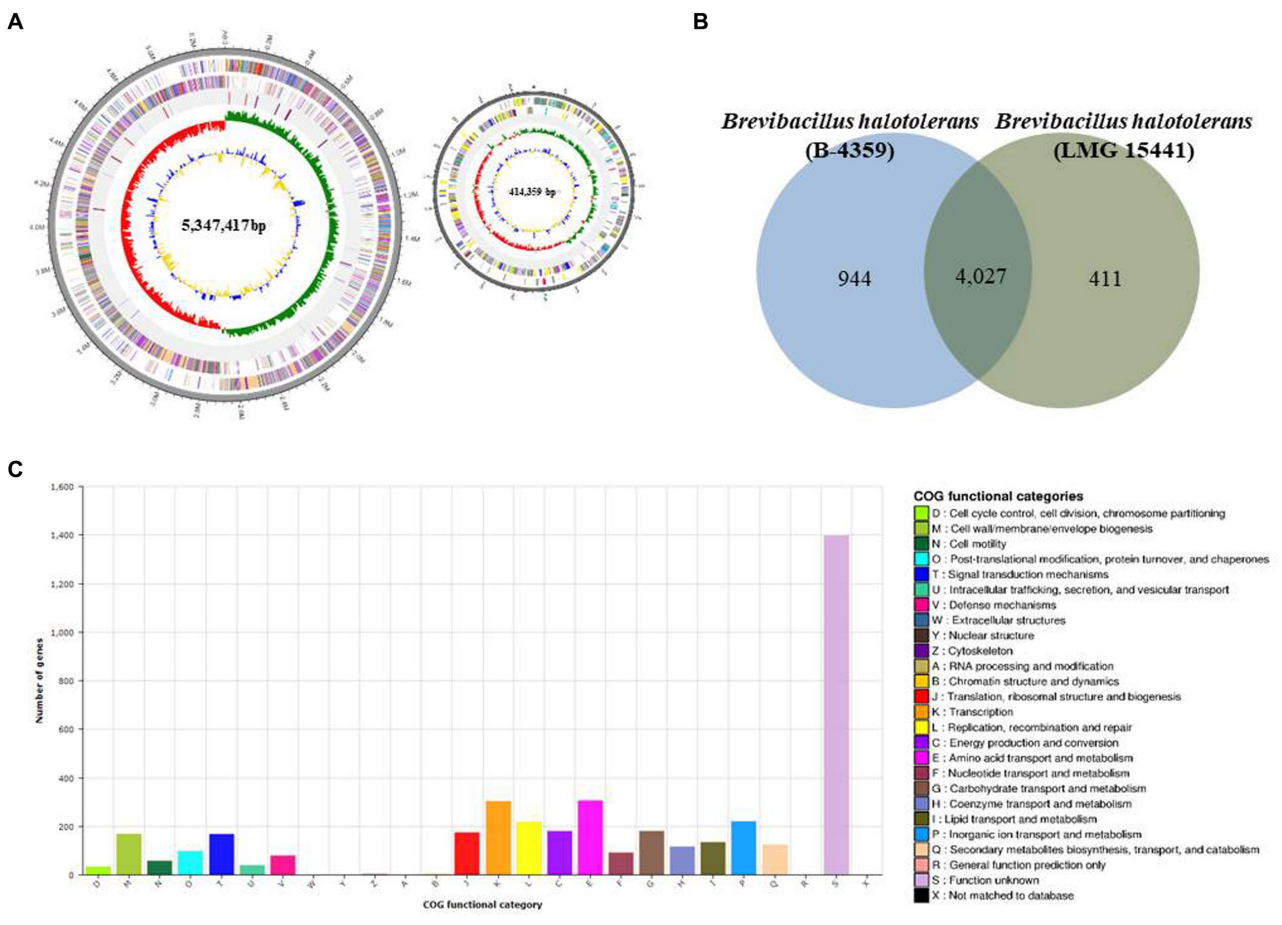
Whole-genome map and COG annotation of *Brevibacillus halotolerans* B-4359. **(A)** Marked characteristics are shown from the outside to the center; coding sequence (CDS) on the forward strand, CDS on the reverse strand, tRNA, rRNA, guanine-cytosine (GC)-content, and GC skew. CDS genome consists of a single circular chromosome that is 5.7 mb in size, and represents one contig from the outer part of the circle; the second circle represents forward, the third represents reverse strain, and the fourth circle represents tRNA and rRNA positions. **(B)** Distribution of orthologous genes in the B-4359 and *B. halotolerans* LMG 15441. The Venn diagram shows the summary of unique SNPs from the total genes of the B-4359. This analysis exploits all CDS of the genomes and is not restricted to the core genome. **(C)** The clusters of orthologous genes (COG) function annotation of B-4359, and distribution of genes in different COG function categories.

PacBio RSII NGS equipment was used at a sequencing depth of 179.344× to obtain complete sequence data for B-4359. The generated raw data were assembled using the HGAP2 protocol to obtain a FASTA file consisting of a single contig. The genome of B-4359 strain consisted of two circular chromosomes of 5,761,776 bp (contig 1, 414,359 bp and contig 2, 5,347,417 bp), with 5,118 predicted protein-coding sequences (CDSs), 36 rRNA genes, 117 tRNA genes, and an average G + C content of 41% based on NCBI Prokaryotic Genomes Automatic Annotation Pipeline (PGAAP) analysis. The first outer circle in the gray region of the genome map represents one contig, second circle represents the forward strand, third circle represents the CDSs on the reverse strand, and fourth circle indicates the tRNA and rRNA positions. The fifth circle represents the GC skew used as a reference point. Values higher than this are indicated in green, whereas lower values are indicated in red. The sixth circle is the GC ratio metric, where values greater than the average GC ratio are expressed in blue and lower values are represented in yellow; the GC skew and GC ratio are expressed at 10 kb intervals (Figure 7A).

The Venn diagram in Figure 7B compares the common CDSs between *B. halotolerans* B-4359 and *B. halotolerans* LMG 15441. This section summarizes the unique protein-encoding genes among all B-4359 genes in total. In total, 4,027 common high-expression gene families were shared between B-4359 and LMG 15441 (Figure 7B), whereas 944 and 411 genes were unique to B-4359 and LMG 15441, respectively. All predicted CDSs of B-4359 were compared with the clusters of orthologous genes (COG) databases to identify homologous amino acid sequences. Each functionally annotated protein was assigned a COG number, representing a class of proteins; then, the proteins were subjected to functional clustering analysis according to the COG function. Totally, 123 and 81 genes were associated with secondary metabolites biosynthesis, transport, catabolism, and defense mechanisms, respectively (Figure 7C). Furthermore, secondary metabolite biosynthesis genes of B-4359 were detected using antiSMASH. A total of 23 biosynthetic gene clusters were identified, of which 8 secondary metabolite gene clusters encoding NRP biosynthesis were identified (Supplementary Table 4). A genome search revealed that strain B-4359 produces several antimicrobial compounds that suppress the growth of fungal pathogens. Five secondary metabolites with >95% similarity were identified using antiSMASH. In gene expression for five secondary metabolite gene clusters using real-time PCR, basiliskamide, and macrobrevin were expressed at a higher level than the other gene clusters (Supplementary Figure 4).

## Discussion

From the *in vitro* screening performed in our study, nine antagonistic bacterial strains were selected from 856 isolates obtained from FBCC, Korea. From these nine isolates, based on *in vitro* antagonistic activity against three fungal pathogens (*C. acutatum*, *F. oxysporum*, and *R. solani*) and three bacterial pathogens (*X. arboricola*, *P. carotovorum*, and *E. pyrifoliae*), and *in vitro* enzyme activity, only one representative isolate *B. halotolerans* B-4359 was selected for further studies. *Brevibacillus* can be found in a variety of environments including soil, seawater, and animal intestines, and it produces various metabolites with antifungal activity that can control plant diseases as BCAs (Yang and Yousef, 2018). Many biological and chemical agents have been used to control bacterial and fungal pathogens in the field (Lee et al., 2017). In recent years, the demand for safe and eco-friendly agricultural products has been increasing (Huh and Kim, 2010). Biological control practices are in high demand as alternatives to synthetic pesticides. Biological control practices are particularly important in organic crop production (Cook, 1993; Yang et al., 2002; Kim and Yun, 2011). Although *Brevibacillus* spp. possessed fewer antagonistic effects on plant pathogens than *Bacillus* spp. and *Pseudomonas* spp., they perform biological control functions against fungal plant pathogens and pests (Joo et al., 2005; Prasanna et al., 2013; Le Han et al., 2022). *B. halotolerans* has recently been reported as a new species with larvicidal activity (Katak et al., 2021). In addition, the abundance of bacteria in the genera *Bacillus*, *Microbacterium*, and *Pseudomonas* have been reported to be effective for biological control, with a positive correlation with *Brevibacillus* sp. (Li et al., 2021). This study describes the biological control effects and characterization of *B. halotolerans* B-4359.

Secondary metabolites, including volatile and non-volatile compounds produced by different bacterial species, play different roles in plants (Rao et al., 2016; Elnahal et al., 2022). The VOCs derived from bacterial sources contribute to disease suppression (Schulz-Bohm et al., 2017). Several *Bacillus* species produce VOCs that exhibit antifungal activities against various phytopathogenic fungi (Zhao et al., 2019; Calvo et al., 2020). Similarly, many other soil bacteria have been displayed to produce VOCs with antifungal effects that contribute to the inhibition of conidia (Garbeva et al., 2011). VOCs produced by *Streptomyces* spp. exhibit antifungal properties against *Rhizoctonia solani*, resulting in plant disease suppression (Cordovez et al., 2015); while VOCs from the tomato rhizosphere bacterium, *Pseudomonas donghuensis* P482, exhibit strong antifungal activity (Ossowicki et al., 2017). These reports are in agreement with our result, where the VOCs derived from strain B-4359 were effective in inhibiting the growth of fungal mycelia of *C. acutatum* using the sandwich plate method. However, the cell-free *CF* of B-4359 inhibited pathogenic mycelial growth to some extent compared to a non-treated control, but not to a significant level. In support of this, Wang et al. (2009) previously reported that the CFs from *Bacillus* spp. exhibited remarkable antifungal activity against several fungal phytopathogens, whereas some other studies reported that the CFs of *B. amyloliquefaciens* AK-0 inhibited the growth and spore germination of *Cylindrocarpon destructans* (Kim et al., 2016).

Our strain B-4359 did not exhibit an inhibitory effect on conidial germination when mixed with conidial suspensions of the fungal pathogen; however, treatment with B-4359 cell suspensions promoted spore germination in comparison with that of non-treated control. But the general phenomenon is that when new biocontrol products are to be developed, strains are mostly selected based on their ability to inhibit our target pathogens under *in vitro* conditions *via* antagonistic effects or *in vitro* suppression of spore germination. Strains with no *in vitro* effect are often eliminated without further testing *in planta*. Our results are supported by a previous report (Besset-Manzoni et al., 2019), where the BCAs have not exhibited antagonistic effects *in vitro* but had good efficacy *in planta*. In contrast, a previous study by Han et al. (2015) stated that the conidial germination of *C. gloeosporioides* was inhibited by four *B. atrophaeus strains*, of which the *B. atrophaeus* strain HM03 suppressed the conidial germination of *C. gloeosporioides* and *C. acutatum*. This antagonistic effect may be owing to the secretion of certain antifungal metabolites by *Bacillus* spp. (Fan et al., 2017). Of note, B-4359 displayed a disease-control effect against anthracnose in red pepper fruits. *Brevibacillus* is known to have a germination inhibitory effect on spores of other fungal phytopathogens, such as *Alternaria* sp., *Fusarium* sp., and *Colletotrichum* sp. (Saber et al., 2003; Chandel et al., 2010; Ahmed, 2017), but such an inhibitory effect was not observed with B-4359 treatment.

Furthermore, BCAs substituted for chemicals in modern agriculture require the ability to stimulate plant growth and ameliorate diseases (Kamil et al., 2018). The plant growth-promoting ability of B-4359 was tested, and the B-4359 suspension treatment promoted plant growth. Plant growth-promoting bacteria with biocontrol abilities can produce secondary metabolites such as antibiotics and phytohormones (Backer et al., 2018). Our results showed that B-4359 produced IAA in addition to effectively inhibiting pathogenic fungi, resulting in promoting plant growth. The ability of bacteria to produce IAA is an important factor that influences plant growth (Chandra et al., 2018). These compounds stimulate root growth, resulting in a larger root surface area that enables plants to access more nutrients from the soil, thereby promoting plant growth (Souza et al., 2013). The application of B-4359 in the field showed an excellent biological control effect in the second investigation. Disease incidence under field conditions is affected by a variety of parameters, such as the nature of pathogenicity, environmental factors, and soil microbes (Wei et al., 2015). Strain B-4359 did not directly antagonize the pathogen *in vitro* or inhibit spore germination; it did induce the expression of the plant systemic resistance marker gene *CaPR1* and the housekeeping gene actin (*ACT*).

Our study further reports the whole-genome sequence of strain B-4359, which consists of chromosome 5,761,776 bp. The B-4359 genome was compared with those of similar species of the same genus using whole-genome analysis. The non-ribosomal peptide (NRP) gene clusters of *Brevibacillus* spp. encode antimicrobial cyclic peptides such as bacillibactin, brevicidine, and brevicidine B (Li Y. X. et al., 2018; Zhao and Kuipers, 2021). In particular, biocontrol-related genes and gene clusters involved in antibiotic resistance could result in differences in biocontrol targets and efficacy between B-4359 and other *Brevibacillus* strains. These results suggest that all other strains could prevent disease through the genes involved in the synthesis of secondary metabolites. The genome of strain B-4359 was investigated for the presence of secondary metabolites using antiSMASH and eight secondary metabolite gene clusters encoding NRP biosynthesis were identified. Among them, siderophore (petrobacterin) and three antibacterial peptides (bogorol A, macrobrevin, brevicidine) were identified as 100% in contig 2; they play a role in the suppression of pathogens by inducing systemic resistance (Schneider et al., 2007). Similarly, in a recent report by Le Han et al. (2022), genomic analysis of the strain *B. halotolerans* 7WMA2 isolated from marine sediments revealed 23 putative biosynthetic secondary metabolite gene clusters responsible for NRP, polyketides, and siderophores.

In conclusion, strain B-4359 obtained from FBCC exhibited remarkable antagonistic activity against several fungal pathogens, including *C. acutatum*, which causes anthracnose in red peppers. B-4359 cell suspensions suppressed red pepper anthracnose *in vitro* despite promoting conidial germination. This strain produced IAA and showed growth-promoting effects on red pepper seedlings. The B-4359 suspension treatment has been demonstrated to control red pepper anthracnose under field conditions in Andong, Gyeongbuk Province, South Korea. Whole-genome sequencing revealed that the core genome of B-4359 was 98.6% similar to that of *B. halotolerans* LMG 15441. The strain was identified as *B. halotolerans* B-4359 based on the whole-genome sequence analysis. The whole-genome sequencing of strain B-4359 revealed antimicrobial secondary metabolites such as bogorol A, basiliskamide A/B, macrobrevin, brevicidine, and tauramamide. Petrobactin siderophore-coding regions have also been identified. Our results indicate that B-4359 may be a promising BCA for controlling phytopathogens in an eco-friendly manner. Future studies will apply proteomic and transcriptomic methods to investigate the signaling pathways involved in the antagonistic effects of secondary metabolites.

## Data availability statement

The datasets presented in this study can be found in online repositories. The names of the repository/repositories and accession number(s) can be found in the article/Supplementary material.

## Author contributions

HK and YL contributed to design and perform the experiments. Y-JH and M-HL were involved in the field experiments. YL performed the RNA sequencing experiment and supervised the project. KB and YJ analyzed the data and wrote the manuscript. All authors contributed to the manuscript and approved the submitted version.

## Conflict of interest

The authors declare that the research was conducted in the absence of any commercial or financial relationships that could be construed as a potential conflict of interest.

## Publisher’s note

All claims expressed in this article are solely those of the authors and do not necessarily represent those of their affiliated organizations, or those of the publisher, the editors and the reviewers. Any product that may be evaluated in this article, or claim that may be made by its manufacturer, is not guaranteed or endorsed by the publisher.
