# Supplementary material for: Identification and characterization of Brevibacillus halotolerans B-4359: a potential antagonistic bacterium against red pepper anthracnose in Korea
Source: Front Microbiol. 2023 Jun 19;14:1200023. doi: 10.3389/fmicb.2023.1200023 (PMC10315534; doi:10.3389/fmicb.2023.1200023)
Supplement: Supplementary file 1 [file Data_Sheet_1.docx]

## Supplementary information

**Identification and characterization of *Brevibacillus halotolerans* B-4359: a potential antagonistic bacterium against red pepper anthracnose in Korea**

Heejin Kim^1^, Younmi Lee^1,2^, Ye-Ji Hwang^3^, Mi-Hwa Lee^3^, Kotnala Balaraju^2^, Yongho Jeon^1^*

^1^Department of Plant Medicals, Andong National University, Andong 36729, Republic of Korea

^2^Agricultural Science & Technology Research Institute, Andong National University, Andong 36729, Republic of Korea

^3^Microbiology Research Department, Nakdonggang National Institute of Biological Resources, Sanju 37242, Republic of Korea

Heejin Kim and Younmi Lee: These two authors contributed equally

***Correspondence**:

Prof. Yongho Jeon

E-mail: [yongbac@andong.ac.kr](mailto:yongbac@andong.ac.kr)

Tel: +82-54-820-5507

Fax: +82-54-820-6320

**Running title:** Characterization of *Brevibacillus halotolerans*

## Supporting information

## Collection of fungal and bacterial pathogens

## Two fungal and three bacterial pathogens were examined in this study. Fungal pathogens, such as *Colletotrichum acutatum* KACC42403, which causes bitter rot in apples, and *Fusarium oxysporum* f. sp*. lycopersici* KACC40043, which causes wilt disease in tomatoes, were obtained from the Korean Agricultural Culture Collection (KACC), Agricultural Microbiology Division, RDA, South Korea. Bacterial pathogens such as *Xanthomonas arboricola* pv*. juglandis* GYUN-39 (a causative agent of bacterial blight in walnuts), *Pectobacterium carotovorum* GYUN-18 (causes soft rot in tomatoes), and *Erwinia pyrifoliae* GYUN-550 (causes black shoot blight in apples) were isolated from infected walnuts, tomatoes, and apple leaves, respectively, in our laboratory. Fungal pathogens were cultured on potato dextrose agar (PDA) plates at 25 °C for 5 d, whereas bacterial pathogens were cultured by plating on tryptic soy agar (TSA) plates and incubated at 28 °C for 48 h.

## *In vitro* screening of antagonistic bacteria

## Totally, 856 bacterial strains were procured from Freshwater Bioresources Culture Collection (FBCC) to screen for *in vitro* antagonistic activity against five plant pathogens (two fungal and three bacterial pathogens as mentioned above). All the FBCC strains were cultured on R2A medium at 28 °C for 48 h. All the bacterial isolates were tested for their antagonistic activity against the fungal and bacterial pathogens using a dual culture plate assay. The fungal pathogens (*C. acutatum* and *F. oxysporum*) were cultured on PDA plates and incubated for 5 d at 25 °C to spread the entire plate with mycelial growth. A mycelial plug (5 mm in diameter) from actively growing fungal pathogens on a PDA plate was removed with a sterile cork borer and placed at the centre of the PDK or R2A agar plates (90 mm diameter). Four streaks were created on the edges of the plates containing four actively growing bacterial suspensions. The inhibition of fungal growth was observed after incubating the plates at 25 °C for 7 d. The antifungal activity was calculated as growth inhibition (%) = [(c−t)/c] × 100 (where ‘c’ is control and ‘t’ is test). To test antagonistic activity against bacterial pathogens, freshly cultured pathogenic bacterial suspensions were spread onto TSA and R2A plates using a sterile spreader, followed by treatment with four different antagonistic bacterial cultures on four sides using a sterile cotton swab. The inhibition zone was measured after incubation of the plates at 28 °C for 48 h. All the experiments were performed twice. All the bacterial isolates were maintained at −80 °C in TSB with glycerol (20%) for long-term storage.

***In vitro* enzyme activities by antagonistic bacteria**

The ability of bacteria to produce various enzymes such as amylase, cellulase, protease, and chitinase was tested under *in vitro* conditions. Amylase production was assessed by inoculating bacterial suspensions in a nutrient agar (NA) medium supplemented with 0.5% soluble starch. Sterile paper discs (6 mm) impregnated with bacterial cell suspensions (10 µl) were plated on solid media, and Petri dishes were incubated at 28 °C for 4 d. After pouring iodine solution (0.3 g iodine and 0.6 g KI/L) on the surface of Petri plates if any clear halo zones around the colonies were observed, they could be considered as a positive reaction for starch degradation by bacteria. Cellulase production was assessed by inoculating bacterial cell suspensions in TSA medium supplemented with 5 g/L carboxymethyl cellulose (CMC). Sterilised filter paper discs (6 mm diameter) impregnated with 10 µL bacterial cell suspensions were placed on Petri dishes on four sides 15–20 mm away from the edge of plates and then incubated at 28 °C for 4 d. After pouring a 0.1% Congo red solution onto the surface of Petri dishes, the solution was retained for 5–10 min. The development of a clear halo around the colonies indicated a positive reaction and the production of cellulase by the bacteria. For protease activity, bacterial isolates (10 µl) on sterile paper discs (6 mm in diameter) were inoculated on gelatin media (i.e., gelatin 5 g, beef extract 3 g, protease peptone 5 g, agar 15 g, distilled water 1,000 mL); plates were then incubated at 28 °C for 4 d. After dispensing the plates with 1% tannic solution for 5 min for staining, they were washed with SDW and protease activity was recorded through the development of a clear zone (halo) around the colonies, indicating that the proteins were hydrolysed by the bacteria. A chitinase activity assay was performed using a modified method developed by Joe et al. (2017). Colloidal chitin was prepared from chitin (HiMedia). Chitin powder (40 g) was slowly added to 300 ml of 12 M HCl and heated for 60 min at 30 °C with vigorous stirring. Chitin was precipitated as a colloidal suspension by adding 2 L of cold SDW at 4–10 °C. After overnight incubation in a refrigerator at 4 °C, the residue was obtained by filtration using a two-layer filter paper. The solution was washed three times until the pH of the suspension was 3.5. Approximately 1 L of tap water in the remaining mass was filtered again using a two-layer filter paper, and the remaining solid (residue) was obtained. Colloidal chitin was used as a substrate. In order to screen bacteria that produce a chitin-degrading enzyme, a medium with the following composition was prepared and used: 6 g Na_2_HPO_4,_ 3 g KH_2_PO_4_, 1 g NH_4_Cl, 0.5 g NaCl, 0.05 g yeast extract, 15 g agar, 1% (w/v) colloidal chitin, and made up to 1 L. The sterile paper disks (6 mm in diameter) impregnated with 10 µl of the suspensions of the candidate strains were placed onto a solid medium, and hydrolytic clearing zones were observed 7 d after incubating the plates at 28 °C.

**Phosphate solubilisation assay**

To determine the ability of bacteria to utilise phosphate *in vitro*, fresh 24-h-old cultured suspensions of all the nine isolates (15 µl) were impregnated on sterile paper discs (6 mm diameter) and placed onto National Botanical Research Institute’s phosphate (NBRIP) agar plates (containing glucose 10 g, Ca_3_(PO_4_)_2_ 5 g, MgCl_2_·6H_2_O 5 g, MgSO_4_·7H_2_O 0.25 g, KCl 0.2 g, and (NH_4_)_2_SO_4_ 0.1 g; pH-7.0 and agar 15 g/L). Clear halo zones were detected after 7 d of incubation at 25 °C. Clear halos indicate the ability of the bacteria to solubilise phosphate. The experiment was repeated once in triplicates.

**Siderophore production assay**

All bacterial strains were assessed for their siderophore-producing ability using the chrome azurol sulfate assay (CAS) developed by Schwyn and Neilands (1987). In brief, CAS solution was prepared as follows: 121 mg CAS was dissolved in 100 ml distilled water and 20 ml of 1 mM ferric chloride (FeCl_3_·6H_2_O) solution was prepared in 10 mM HCl. This solution was added to 20 ml hexadecyl trimethyl ammonium bromide (HDTMA) solution with stirring. An HDTMA solution was prepared by mixing 729 mg of HDTMA in 400 ml of distilled water. The CAS-HDTMA solution was sterilised prior to use. CAS agar plates were prepared by mixing 100 ml CAS reagent with 900 ml sterilised LB agar medium. Freshly cultured bacterial suspensions (15 µl) were spotted on sterile paper discs (6 mm diameter) and placed onto CAS agar plates and incubated at 25 °C for 7 d. The appearance of an orange or yellow zone around the disc indicated siderophore production. The experiment was performed twice in triplicate.

**Auxin and Gibberellin detection assays**

To detect the presence of auxins in bacteria, the method developed by Darkoh et al (2015) was used. In brief, bacterial cell suspensions were inoculated into the LB medium containing 5 mM L-tryptophan and incubated at 25 °C for 2 d under shaking conditions at 180 rpm. The bacterial culture was centrifuged at 15000 × *g* under cool conditions (4 °C) for 10 min and filtered to collect the supernatant. The supernatant (0.2 ml) was mixed with 1.0 ml of Kovac’s reagent (Sigma-Aldrich, St. Louis, MO), incubated for 30 min at 27 °C, and the absorbance was read at 560 nm. The experiment was performed twice in triplicate.

To detect the presence of gibberellin, bacterial suspensions were inoculated into freshly prepared LB broth and incubated at 25 °C for 3 d under shaking conditions (180 rpm). The supernatant was collected after centrifuging the cell suspensions at 15000 × *g* and 4 °C for 30 min and filtration. The filtered supernatant of approximately 1.0 ml was mixed with 8.0 ml of 3.74 M HCl and incubated at 27 °C for 5 min. The absorbance was read at 254 nm, and the concentration of gibberellin was determined by comparison with the standard curve. The experiment was repeated once in triplicate.

**References**

Schwyn, B., and Neilands, J. B. (1987). Universal chemical assay for the detection and determination of siderophores. *Anal. Biochem*. 160, 47–56.

Darkoh, C., Chappell, C., Gonzales, C., and Okhuysen, P. (2015). A rapid and specific method for the detection of indole in complex biological samples. *Appl. Environ. Microbiol*. 81, 8093–8097.

**Supplementary Tables**

### Supplementary Table 1. Treatment schedule and details of field trial

| **Treatment/Date** | **6/24** | **7/6** | **7/15** | **7/26** | **8/5** | **8/16** |
| --- | --- | --- | --- | --- | --- | --- |
| Control | - | - | - | - | - | - |
| Chemical control | Bion-M | Propineb | Pyraclostrobin | Chlorothaloniil+ Difenoconazole | Dithianon+ Pyraclostrobin | Prochloraz+ Tebuconazole |
| Pyraclostrobin | Pyraclostrobin | Pyraclostrobin | Pyraclostrobin | Pyraclostrobin | Pyraclostrobin | Pyraclostrobin |
| PI B-4359 | Bion-M | Propineb | Pyraclostrobin | Chlorothaloniil+ Difenoconazole | Dithianon+ Pyraclostrobin | Prochloraz+ Tebuconazole |
| FS B-4359 | B-4359 | B-4359 | B-4359 | B-4359 | B-4359 | B-4359 |
| FS+SD B-4359 | B-4359 | B-4359 | B-4359 | B-4359 | B-4359 | B-4359 |

# Supplementary Table 2. Antifungal activity (percent inhibition) of each isolate against two fungal pathogens

| Pathogen | FBCC | Species | Mycelium growth Inhibition (%) | Inhibition expression |
| --- | --- | --- | --- | --- |
| *Colletotrichum acutatum* | B-1662 | *Streptomyces sporoverrucosus* | 81.0 | +++ |
|  | B-4371 | *Streptomyces virginiae* | 71.4 | +++ |
|  | B-4370 | *Streptomyces virginiae* | 68.3 | +++ |
|  | B-4366 | *Brevibacillus halotolerans* | 61.9 | +++ |
|  | B-248 | *Paenibacillus terrae* | 58.7 | ++ |
|  | B-4339 | *Chromobacterium alkanivorans* | 55.6 | ++ |
|  | B-4359 | *Brevibacillus halotolerans* | 52.4 | ++ |
|  | B-1661 | *Bacillus altitudinis* | 52.4 | ++ |
|  | B-4343 | *Chromobacterium alkanivorans* | 52.4 | ++ |
|  | B-4407 | *Streptomyces angustmyceticus* | 52.4 | ++ |
|  | B-1625 | *Bacillus altitudinis* | 49.2 | ++ |
|  | B-4346 | *Streptomyces angustmyceticus* | 44.8 | ++ |
|  | B-4434 | *Streptomyces hygroscopicus* subsp*. ossamyceticus* | 44.8 | ++ |
|  | B-1635 | *Bacillus altitudinis* | 42.9 | ++ |
|  | B-1636 | *Bacillus altitudinis* | 42.9 | ++ |
|  | B-1909 | *Pseudomonas fluorescens* | 41.4 | ++ |
|  | B-1613 | *Pseudomonas juntendi* | 37.9 | + |
|  | B-1617 | *Janthinobacterium lividum* | 37.9 | + |
|  | B-1618 | *Bacillus aryabhattai* | 37.9 | + |
|  | B-1619 | *Pseudomonas koreensis* | 34.5 | + |
|  | B-1621 | *Acidovorax facilis* | 34.5 | + |
|  | B-1612 | *Pseudomonas koreensis* | 31.0 | + |
|  | B-1791 | *Bacillus altitudinis* | 31.0 | + |
|  | B-1610 | *Acidovorax delafieldii* | 27.6 | + |
|  | B-1798 | *Bacillus altitudinis* | 27.6 | + |
|  | B-1849 | *Klugiella xanthotipulae* | 27.6 | + |
|  | B-4375 | *Streptomyces angustmyceticus* | 27.6 | + |
|  | B-4392 | *Chitinimonas viridis* | 27.6 | + |
|  | B-1615 | *Bacillus megaterium* | 24.1 | + |
|  | B-1657 | *Bacillus proteolyticus* | 24.1 | + |
|  | B-4352 | *Chromobacterium alkanivorans* | 20.7 | + |
|  | B-1607 | *Vogesella indigofera* | 20.7 | + |
|  | B-1616 | *Rhodococcus aetherivorans* | 20.7 | + |
|  | B-1641 | *Pseudoduganella eburnea* | 20.7 | + |
|  | B-1808 | *Pseudomonas gessardii* | 20.7 | + |
|  | B-1908W/Y | *Flavobacterium succinicans* | 20.7 | + |
|  | B-4364 | *Caulobacter vibrioides* | 20.7 | + |
|  | B-1620 | *Bacillus aryabhattai* | 17.2 | + |
|  | B-1637 | *Pseudomonas knackmussii* | 17.2 | + |
|  | B-1638 | *Pseudoduganella danionis* | 17.2 | + |
|  | B-1642 | *Bacillus megaterium* | 17.2 | + |
|  | B-1645 | *Bacillus megaterium* | 17.2 | + |
|  | B-1659 | *Psychrobacillus lasiicapitis* | 17.2 | + |
|  | B-4363 | *Pseudomonas veronii* | 17.2 | + |
| *Fusarium oxysporum* | B-4370 | *Streptomyces virginiae* | 71.1 | +++ |
|  | B-4371 | *Streptomyces virginiae* | 68.4 | +++ |
|  | B-4366 | *Brevibacillus halotolerans* | 68.4 | +++ |
|  | B-1662 | *Streptomyces sporoverrucosus* | 63.2 | +++ |
|  | B-4359 | *Brevibacillus halotolerans* | 47.7 | ++ |
|  | B-324 | *Staphylococcus epidermidis* | 41.5 | ++ |
|  | B-336 | *Arthrobacter bambusae* | 41.5 | ++ |
|  | B-4407 | *Streptomyces angustmyceticus* | 38.5 | + |
|  | B-4346 | *Streptomyces angustmyceticus* | 35.4 | + |
|  | B-297 | *Lelliottia jeotgali* | 35.4 | + |
|  | B-4375 | *Streptomyces angustmyceticus* | 32.3 | + |
|  | B-1836 | *Polynucleobacter yangtzensis* | 29.2 | + |
|  | B-320 | *Sphingomonas paucimobilis* | 29.2 | + |
|  | B-248 | *Paenibacillus terrae* | 26.2 | + |
|  | B-392 | *Caballeronia udeis* | 26.2 | + |

+: < 40.0 % inhibition; ++: ≥ 40.0% and < 60.0; +++: ≥60%

# Supplementary Table 3. Antibacterial activity (zone of inhibition in millimetres) of each isolate against the three bacterial pathogens

| Pathogen | FBCC | Species | Inhibition (mm) |  |
| --- | --- | --- | --- | --- |
| *Xanthomonas arboricola* | B-248 | *Paenibacillus terrae* | 6 | ++ |
|  | B-385 | *Bacillus mycoides* | 5 | ++ |
|  | B-386 | *Bacillus mycoides* | 5 | ++ |
|  | B-395 | *Pseudarcobacter venerupis* | 5 | ++ |
|  | B-251 | *Elizabethkingia anophelis* | 4 | + |
|  | B-210 | *Flavobacterium aquatile* | 3 | + |
|  | B-283 | *Paenibacillus terrae* | 3 | + |
|  | B-306 | *Paenibacillus pabuli* | 3 | + |
|  | B-391 | *Caballeronia udeis* | 3 | + |
|  | B-4359 | *Brevibacillus halotolerans* | 2 | + |
|  | B-4366 | *Brevibacillus halotolerans* | 2 | + |
| *Pectobacterium carotovorum* | B-394 | *Lactobacillus plantarum* subsp*. argentoratensis* | 11 | +++ |
|  | B-389 | *Lactobacillus plantarum* subsp*. argentoratensis* | 9 | ++ |
|  | B-390 | *Lactobacillus plantarum* subsp*. argentoratensis* | 9 | ++ |
|  | B-4359 | *Brevibacillus halotolerans* | 6 | ++ |
|  | B-4366 | *Brevibacillus halotolerans* | 5 | ++ |
|  | B-1662 | *Streptomyces sporoverrucosus* | 4 | + |
|  | B-1693 | *Pseudomonas jessenii* | 4 | + |
|  | B-360 | *Pseudomonas koreensis* | 2 | + |
|  | B-4362 | *Chromobacterium alkanivorans* | 2 | + |
|  | B-386 | *Bacillus mycoides* | 1 | + |
|  | B-395 | *Pseudarcobacter venerupis* | 1 | + |
|  | B-1665 | *Pseudomonas lactis* | 1 | + |
|  | B-4352 | *Chromobacterium alkanivorans* | 1 | + |
| *Erwinia pyrifoliae* | B-4359 | *Brevibacillus halotolerans* | 17 | +++ |
|  | B-4352 | *Chromobacterium alkanivorans* | 12 | +++ |
|  | B-1909 | *Pseudomonas fluorescens* | 4 | + |
|  | B-283 | *Paenibacillus terrae* | 1.5 | + |
|  | B-4366 | *Brevibacillus halotolerans* | 1 | + |
|  | B-248 | *Paenibacillus terrae* | 1 | + |

+: clear zone ≥ 1 mm; ++: ≥5 and < 10; +++: ≥10

**Supplementary Table 4. Predicted secondary metabolite gene clusters identified using antiSMASH in *B. halotolerans* B-4359**

| Region | Type | From | To | Most similar known cluster | | Similarity (%) | MIBiG accession | gene cluster from organisms |
| --- | --- | --- | --- | --- | --- | --- | --- | --- |
| Region 2.1 | RRE-containing | 390071 | 410883 | pyoverdin | NRP | 1 | BGC0000413 | *Pseudomonas protegens* Pf-5 |
| Region 2.2 | cyclic-lactone-autoinducer | 774093 | 793951 |  |  |  |  |  |
| Region 2.3 | siderophore | 895504 | 909203 | petrobactin | Other | 100 | BGC0000942 | *Bacillus anthracis* str. Ames |
| Region 2.4 | NRPS-like,NRPS | 972357 | 1066647 | bogorol A | NRP | 100 | BGC0001532 | *Brevibacillus laterosporus* |
| Region 2.5 | transAT-PKS,PKS-like,T3PKS,NRPS,RiPP-like,NRPS-like | 1848796 | 1990419 | aurantinin B / aurantinin C / aurantinin D | Polyketide | 25 | BGC0001520 | *Bacillus subtilis* |
| Region 2.6 | NRPS | 2038848 | 2088689 | zwittermicin A | NRP + Polyketide | 7 | BGC0001059 | *Bacillus cereus* |
| Region 2.7 | transAT-PKS | 2259670 | 2325611 | basiliskamide A / basiliskamide B | Polyketide | 95 | BGC0000172 | *Brevibacillus laterosporus* PE36 |
| Region 2.8 | RRE-containing | 2412103 | 2431117 |  |  |  |  |  |
| Region 2.9 | transAT-PKS,PKS-like,T3PKS | 2469907 | 2575745 | macrobrevin | Polyketide:Trans-AT type I | 100 | BGC0001470 | *Brevibacillus* sp. Leaf182 |
| Region 2.10 | NRPS,T1PKS,terpene | 2662828 | 2797615 | tauramamide | NRP | 36 | BGC0001796 | *Brevibacillus laterosporus* DSM 25 |
| Region 2.11 | NRPS,transAT-PKS | 2801054 | 2870353 | chejuenolide A / chejuenolide B | Polyketide | 7 | BGC0001543 | *Hahella chejuensis* |
| Region 2.12 | NRPS | 2881128 | 2961651 |  |  |  |  |  |
| Region 2.13 | NRPS | 2973402 | 3061769 | brevicidine | NRP | 100 | BGC0001536 | *Brevibacillus laterosporus* |
| Region 2.14 | NRPS,transAT-PKS | 3102710 | 3200348 | octapeptin C4 | NRP | 11 | BGC0001715 | *Bacillus circulans* |
| Region 2.15 | T3PKS | 3362425 | 3403480 |  |  |  |  |  |
| Region 2.16 | NRPS | 3492278 | 3534546 | tauramamide | NRP | 18 | BGC0001796 | *Brevibacillus laterosporus* DSM 25 |
| Region 2.17 | NRPS | 3562111 | 3608038 | tyrocidine | NRP | 18 | BGC0000452 | *Brevibacillus brevis* NBRC 100599 |
| Region 2.18 | cyclic-lactone-autoinducer | 3646986 | 3667507 |  |  |  |  |  |
| Region 2.19 | phosphonate | 3907604 | 3948491 |  |  |  |  |  |
| Region 2.20 | NRPS | 4222438 | 4317386 | paenibactin | NRP | 33 | BGC0000401 | *Paenibacillus elgii* B69 |
| Region 2.21 | NRPS | 4353367 | 4401001 |  |  |  |  |  |
| Region 2.22 | RiPP-like | 4526698 | 4536164 |  |  |  |  |  |
| Region 2.23 | LAP,RiPP-like | 4663194 | 4686740 |  |  |  |  |  |


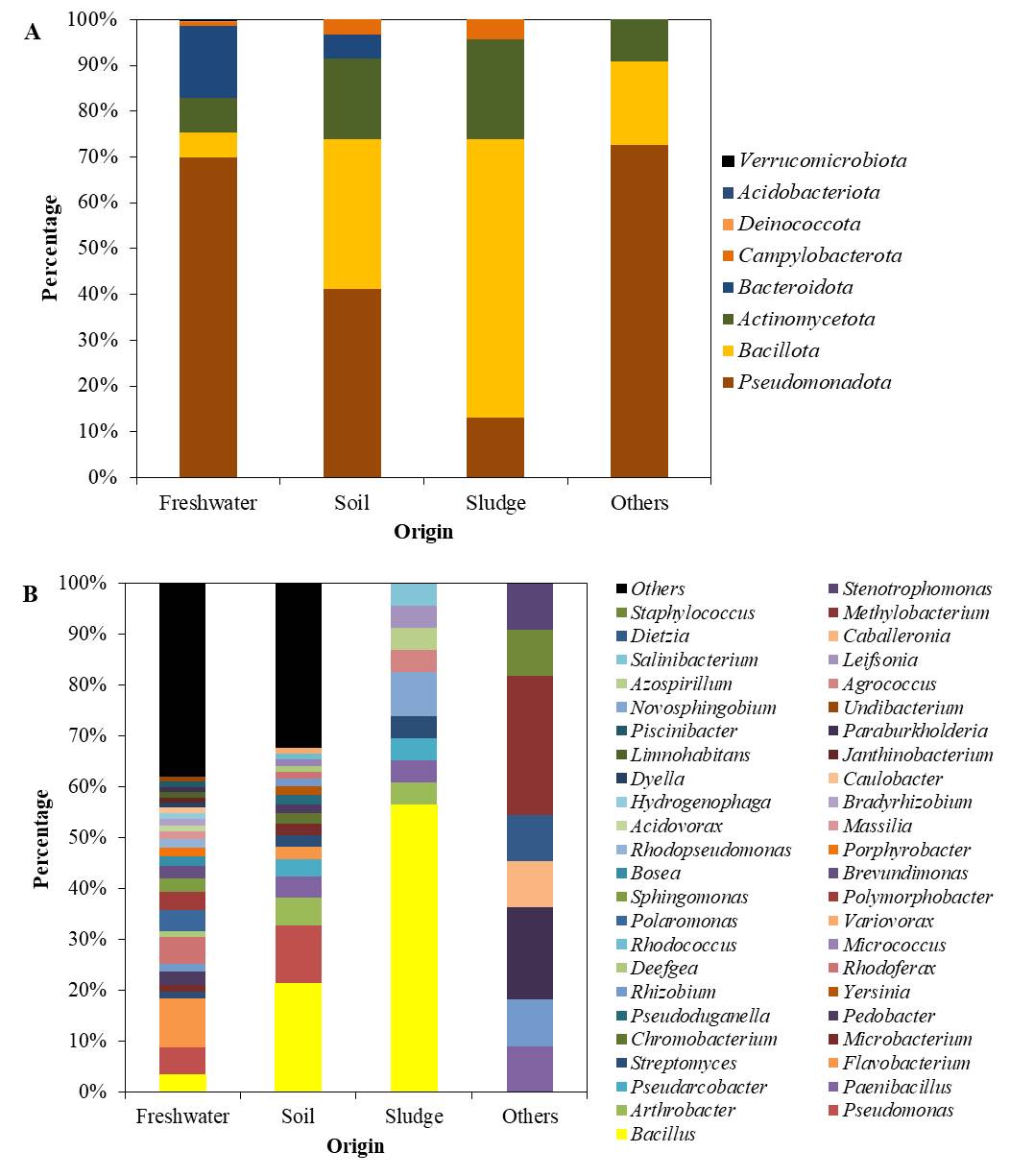
**Supplementary Figures**

**Supplementary Figure 1. Bacterial distribution obtained from FBCC isolates. (**A) Phylum distribution and (B) Genus distribution of the bacterial strains obtained from FBCC. Bacterial strains were 490, 332, 23, and 11 in freshwater, soil, sludge, and others, respectively.


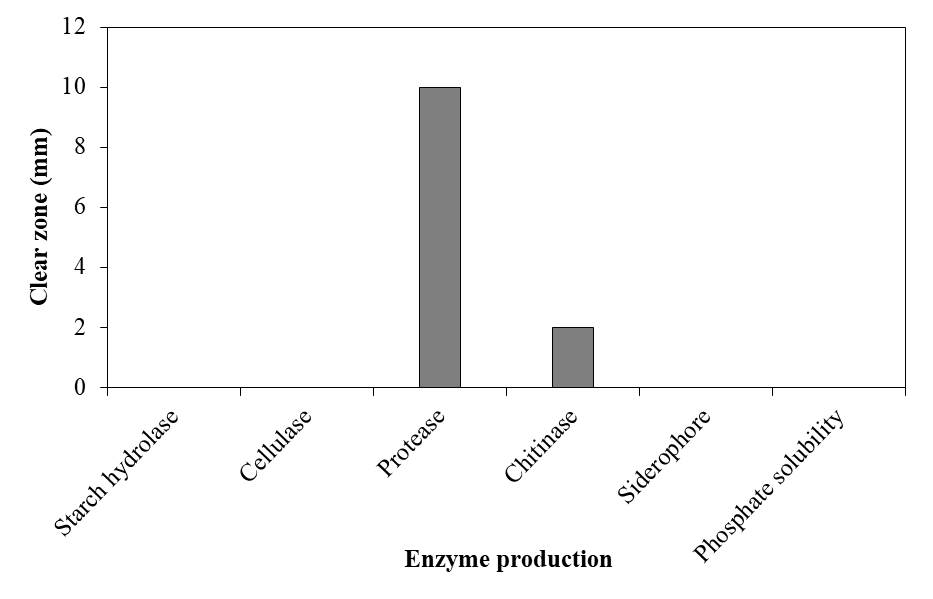


**Supplementary Figure 2. *In vitro* production of enzymes, siderophore production, and phosphate solubility by *B. halotolerans* B-4359**. The experiment was performed at least two times in triplicate per treatment.


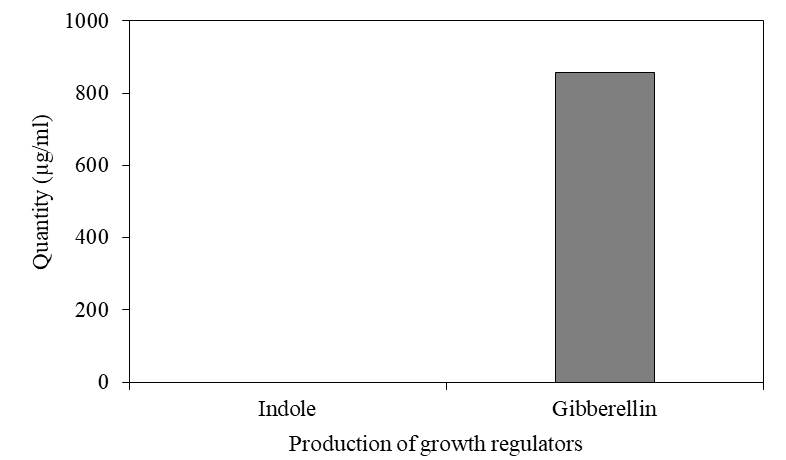


**Supplementary Figure 3. *In vitro* indole and gibberellin production by *B. halotolerans* B-4359**. Only B-4359 showed more than 800 µg/ml gibberellin production. The experiment was performed at least two times in triplicate per treatment.


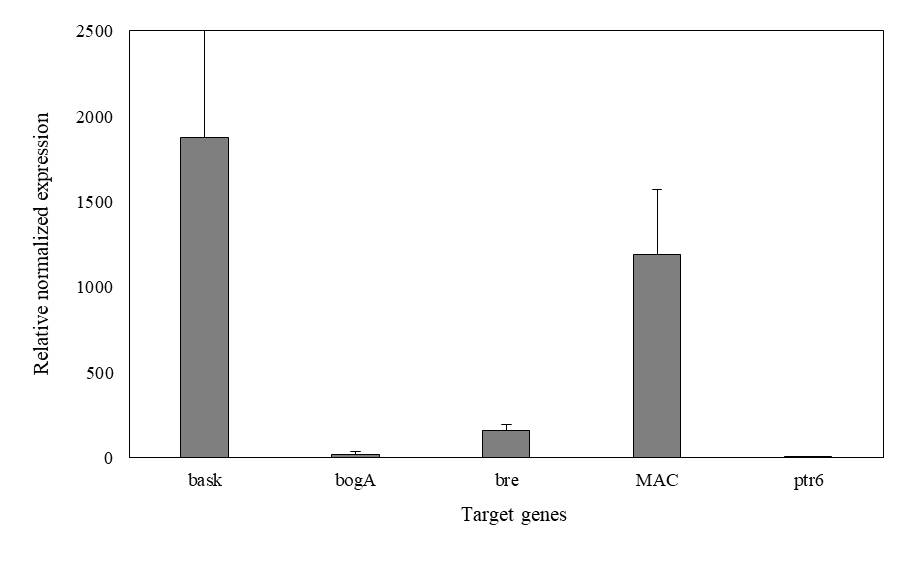


**Supplementary Figure 4.** Gene expression analysis of secondary metabolites from the *B. halotolerans* B-4359 genome using a real-time PCR. MAC and bask genes showed higher gene expression in comparison with other secondary metabolites genes.
